# Alzheimer’s Disease Neurofibrillary Pathophysiology: PTPRD- and Flavonol-Modified Mechanisms, Prevention and Therapeutics

**DOI:** 10.3390/biomedicines14102210

**Published:** 2026-09-29

**Authors:** George R. Uhl, Joungil Choi, Balaji Kannan, Ian Henderson, Emily Hess, Hanna Rogers, Darcy McFarland, Hadley Dowell, Sean Nolan, Kevin Schultz

**Affiliations:** 1 Department of Neurology, School of Medicine, University of Maryland, 4A150a, VAMHCS, 10 N Greene St., Baltimore, MD 21201, USA; 2 Department of Pharmacology, School of Medicine, University of Maryland, Baltimore, MD 21201, USA; 3 Neurology Service, VA Maryland Healthcare System, 10 N Greene St., Baltimore, MD 21201, USA; 4 Research Service, VA Maryland Healthcare System, 10 N Greene St., Baltimore, MD 21201, USA; 5 Department of Internal Medicine, School of Medicine, University of New Mexico, Albuquerque, NM 87106, USA; 6 Department of Chemistry, Goucher College, Towson, MD 21204, USA; emily.hess@goucher.edu (E.H.); seannolan702@gmail.com (S.N.); kevin.schultz@goucher.edu (K.S.)

**Keywords:** GSK3, PTPRD-positive allosteric modulators, flavonols, neurofibrillary tangles

## Abstract

A variety of evidence now supports two interrelated hypotheses concerning Alzheimer’s disease (AD) neurofibrillary pathology (NFT), its prevention and treatment. *The first hypothesis:* the receptor type protein tyrosine phosphatase D (PTPRD) plays a key role in AD NFT pathogenesis by modulating activities of neuronal glycogen synthase kinase 3s (GSK3s) in hyperphosphorylating tau. *The second hypothesis:* positive allosteric modulation of PTPRD’s downregulation of GSK3s by flavonols and improved flavonol analogs provides a novel path to prevention and treatment of AD. We review the convergence of evidence from human, mouse, in vitro and in silico studies that enhances the likely translational relevance of these hypotheses.

## 1. AD and Two Interrelated Hypotheses

**Figure 1:**
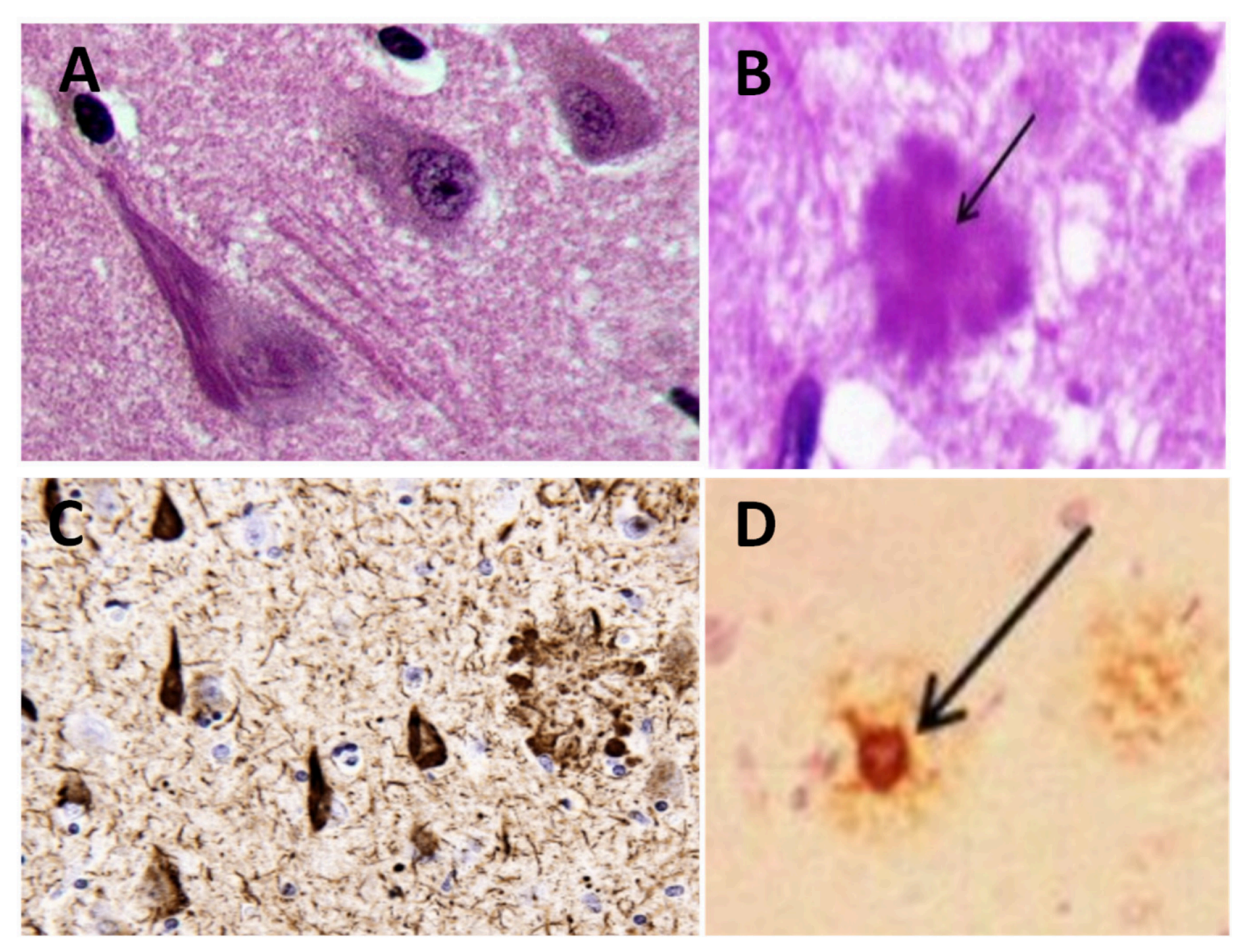
Cardinal AD pathologies. Neurofibrillary tangles (**A**,**C**) and senile plaques. (**B**,**D**) Arrows point to plaque cores. (**A**,**B**) H&E stain. (**C**,**D**) Stains for hyperphosphorylated tau and amyloid Aβ, respectively.

The extent of Alzheimer’s disease (AD) dementia correlates well with the density of neurofibrillary tangles in cerebral cortical and hippocampal neurons [1,2,3]. Pathologically hyperphosphorylated tau (microtubule-associated protein tau) is the major component of these tangles (Figure 1). AD brains also contain senile plaques (Figure 1). Large contributions from amyloid Aβ to these plaques make Aβ a prime target of current disease-modifying therapeutics for AD [4,5].

Overall AD vulnerability results from genetic and environmental influences. Twin datasets document large contributions from additive genetic influences on AD vulnerability [6,7]. Molecular genetic studies support large influences of variation at the apolipoprotein E (ApoE) locus on AD vulnerability and on the densities of neurofibrillary tangles and senile plaques [8,9]. Variation at the receptor-type protein tyrosine phosphatase (PTPRD) locus provides a selective influence on the densities of AD neurofibrillary pathology (NFT) [8] (*see below*).

Environmental influences have also been associated with vulnerability to developing AD. In meta-analyses, individual differences in diet provide some of the largest environmental contributions to individual differences in AD vulnerability [10,11,12].

We now assemble evidence that supports two interrelated hypotheses that have implications for understanding, preventing and treating AD. *First*, PTPRD plays a key role in the pathogenesis of AD neuronal neurofibrillary neuropathology by modulating tau hyperphosphorylation [13,14]. Much of this effect of PTPRD is likely to result from its role in modulating the neuronal activities of the key tau hyperphosphorylating kinases, glycogen synthase kinase 3s (GSK3 α and β) [13]. *Second*, flavonols and improved flavonol analogs provide positive allosteric modulation (PAM) of PTPRD’s dephosphorylation and downregulation of GSK3s [13]. Flavonol analogs thus provide a novel path to the prevention and treatment of AD, especially in those at higher genetic risk.

The evidence that now supports these two hypotheses and their implications for prevention and treatment arises from a variety of diverse approaches. We have contributed to this growing body of evidence and assembled the work of others from repeated search engine and literature searches since we first articulated an early version of these hypotheses [13]. This evidence has not been comprehensively assembled or contextualized. There is limited understanding of PTPRD within much of the community of clinicians and scientists who have interests in AD. We thus discuss PTPRD, GSK3s, flavonols, evidence that supports our two hypotheses, the contexts in which this evidence was derived, the strengths and limitations of the data and the implications of our hypotheses for AD prevention and treatment. We note the implications of this work for intellectual property owned by the US Veterans Affairs Administration. We list abbreviations used herein at the end of this paper.

## 2. PTPRD

**Figure 2:**
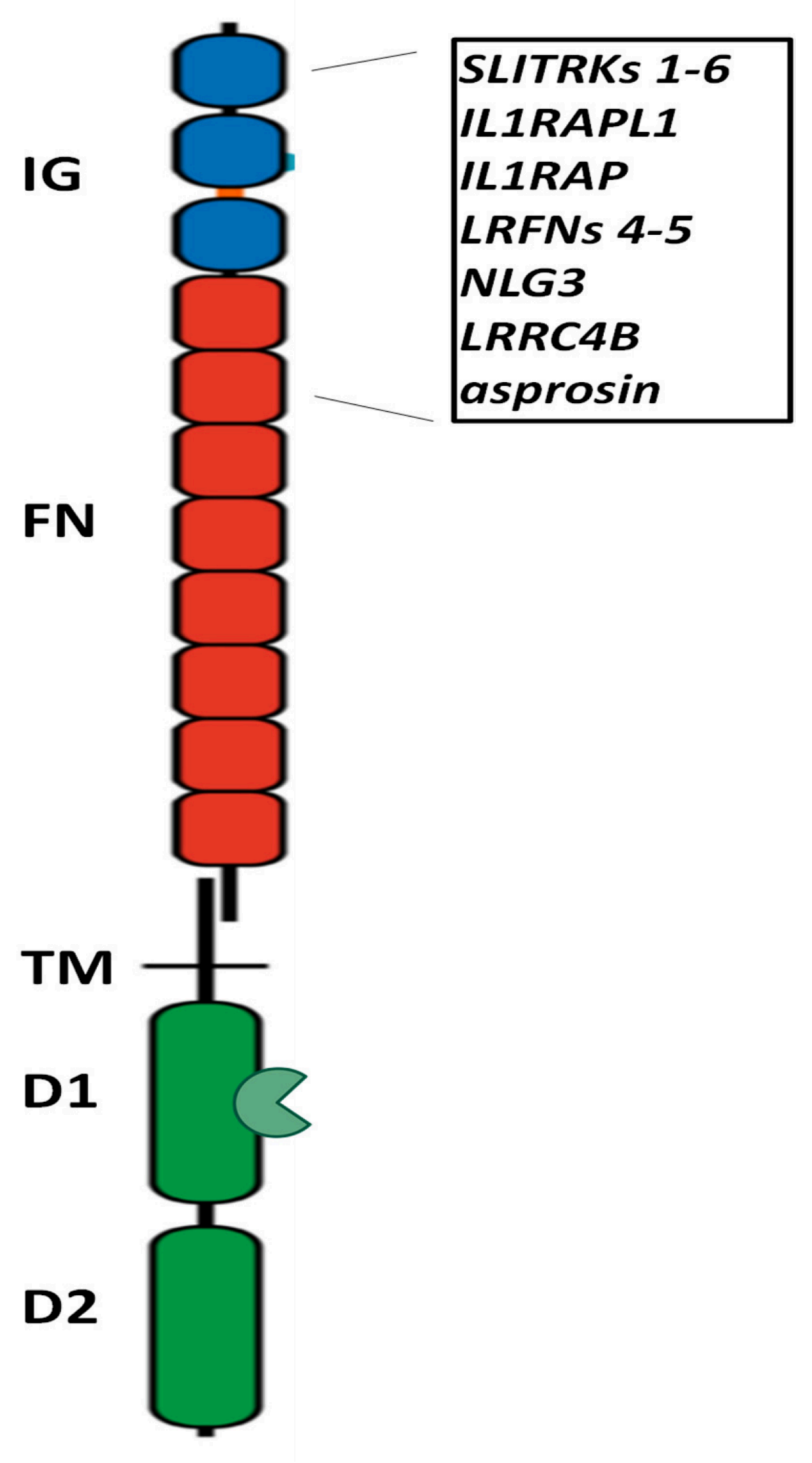
PTPRD. Extracellular immunoglobulin (IG) and fibronectin III (FN), transmembrane (TM) and intracellular D1 phosphatase and D2 domains. Box lists likely PTPRD ligands.

PTPRD is a largely neuronal, largely presynaptic single-transmembrane-domain protein that likely transduces signals from extracellular binding partners to change the activities of its intracellular protein tyrosine phosphatase [15,16] (Figure 2). It is thus likely to change its intracellular phosphatase activity in response to extracellular binding partners that are expressed on the surfaces of adjacent neurons or glia, including the interleukin I receptor-associated, slitrk, neuroligin, leucine-rich repeat and fibronectin type-III domain-containing and leucine-rich repeat-containing proteins or soluble ligands that include the orexigenic hormone asprosin [13,17]. Figure 2 notes PTPRD’s immunoglobulin and fibronectin III extracellular domains, likely binding partners that recognize specific sequences in these domains, the site where furin cleavage during Golgi processing of its proprotein requires its assembly with disulfide bonds, the transmembrane domain, the catalytically active D1 intracellular domain, and the catalytically inactive intracellular D2 domain. PTPRD can dephosphorylate a number of phosphotyrosine phosphoprotein substrates [13]. In this review, we focus our discussion on its ability to dephosphorylate the glycogen synthase kinase 3s (α and β) in ways that downregulate their activities (*below*).

## 3. PTPRD Genetics, Genomics and Pharmacological Confirmation

### 3.1. Human Genetics and Genomics

**Figure 3:**
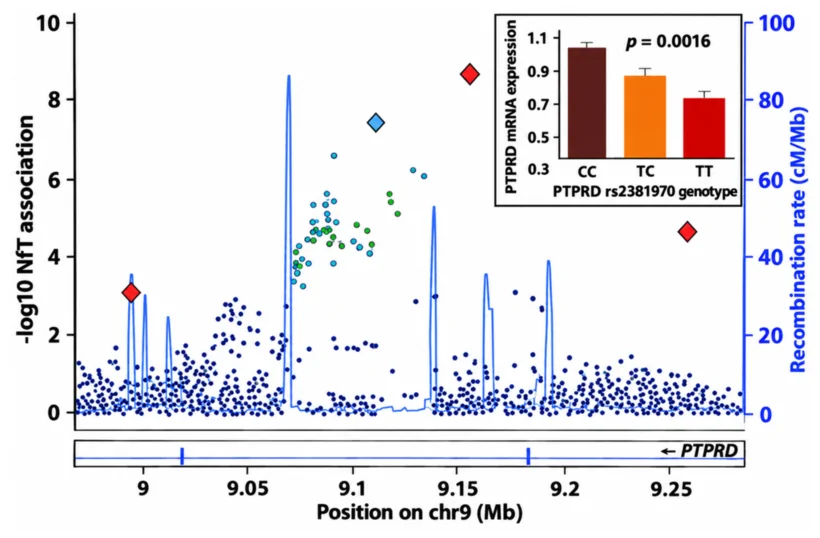
Genetic associations between common PTPRD genomic makers, densities of neurofibrillary tangles in AD brains and levels of PTPRD expression in normal brains. X axis: distance along the chromosome 9p region that harbors PTPRD’s exons 9 and 10 (vertical hash marks) and surrounding introns. Y axes: −log *p* for (*left*) associations of genomic markers (higher value = stronger association) with densities of neurofibrillary tangles (dark, cyan and green; (*right*) association with PTPRD mRNA levels in control brains. Associations for rs560380 (NFTs) and rs2381970 (mRNA levels) remain significant after conservative Bonferroni correction for the total number of SNPs tested (see ref. [3,8]). *Inset:* relative levels of PTPRD mRNA expression in brains from individuals with rs2381970 SNP genotypes CC, TC, and TT (ref. [3]).

Our attention was drawn to the relationship between PTPRD and AD neurofibrillary pathology by genome-wide association (GWAS) work that assessed genomic associations with neuropathological features found in the brains of 909 carefully followed study participants [8]. This work identified a strong association between brain densities of neurofibrillary tangles and common genomic variants in 5′ introns of the PTPRD gene (Figure 3). There was specificity: PTPRD was not associated with densities of senile plaques. The *p* value for this association survived conservative, Bonferroni correction for the large number of repeated comparisons in this study. We were especially intrigued by this finding since NFT densities were associated with genomic variants in the region of the PTPRD gene where we had previously identified associations with the level of expression of PTPRD mRNA in postmortem cortex samples from 119 individuals [3] (Figure 3). Common PTPRD allelic variation thus provides the second strongest genetic influence on the densities of neurofibrillary tangles in a strong GWAS study and confers about 50% individual differences in levels of brain PTPRD expression.

*Context, strengths and weaknesses:* There are modest PTPRD associations with vulnerability to AD in several datasets [8,18]. Common 5′ PTPRD variants display associations with language, memory, executive function and memory test results in several tested cohorts [19]. A human with a very rare absence of PTPRD expression displayed severe deficits in cognitive function [20]. However, variation in PTPRD has not been strongly associated with vulnerability to AD in large meta-analyses of GWAS studies [21]. Variation in PTPRD thus provides only one of many contributors to overall AD pathogenesis.

PTPRD is genetically associated with the densities of neocortical tangles and with the Braak staging of tangles, which is largely based on their location within the brain in [8]. However, there was no significant association with Braak stage in [22]. Densities of tangles may be more clinically relevant than their location. There are strong correlations between individual differences in cognitive function and individual differences in densities of neocortical tangles even *within* a single Braak stage, for example [23].

If the 5′ region of the PTPRD gene contains common variants that are important for the levels of expression of this gene, we might expect associations between variation in this region and other human traits and disorders. Common genomic variation in this region is strongly associated with vulnerability to restless legs syndrome [24] and more modestly associated with addiction-related phenotypes [15].

Our studies of postmortem human brains used controls for pathology and for the quality of the extracted RNA [3]. However, mRNAs from postmortem brain can be labile, especially when tissue integrity is disrupted [25].

Overall, results from human genetic studies that identify effects of common PTPRD genomic variation on densities of neurofibrillary pathology in AD brains provide substantial prior probabilities for the results of studies of mouse models of AD. These priors are enhanced by the extent to which mouse models accurately reflect the level-of-expression differences that are driven by the human variation in PTPRD that is associated with AD NFT densities.

### 3.2. Mouse Genetic and Pharmacologic Models

There is substantial validation of these human PTPRD genetic associations from studies of heterozygous knockout mice with reduced levels of expression of PTPRD and from studies of pharmacological inhibition of PTPRD using our PTPRD inhibitor pentilludin [26].

3xTg-AD/PTPRD+/− mice add a 50% reductions in PTPRD expression to the 3xTg-AD mouse model, providing variation within the range of the differences in levels of PTPRD expression that are driven by common variation in 5′ aspects of the human PTPRD gene [3]. 3xTg-AD mice lack presenilin and overexpress amyloid precursor and tau protein variants that lead to familial AD and frontotemporal dementias, respectively [27,28]. 3xTg-AD mice develop hyperphosphorylated tau and AD-like neurofibrillary pathology over a well-characterized two-year time course [28].

**Figure 4:**
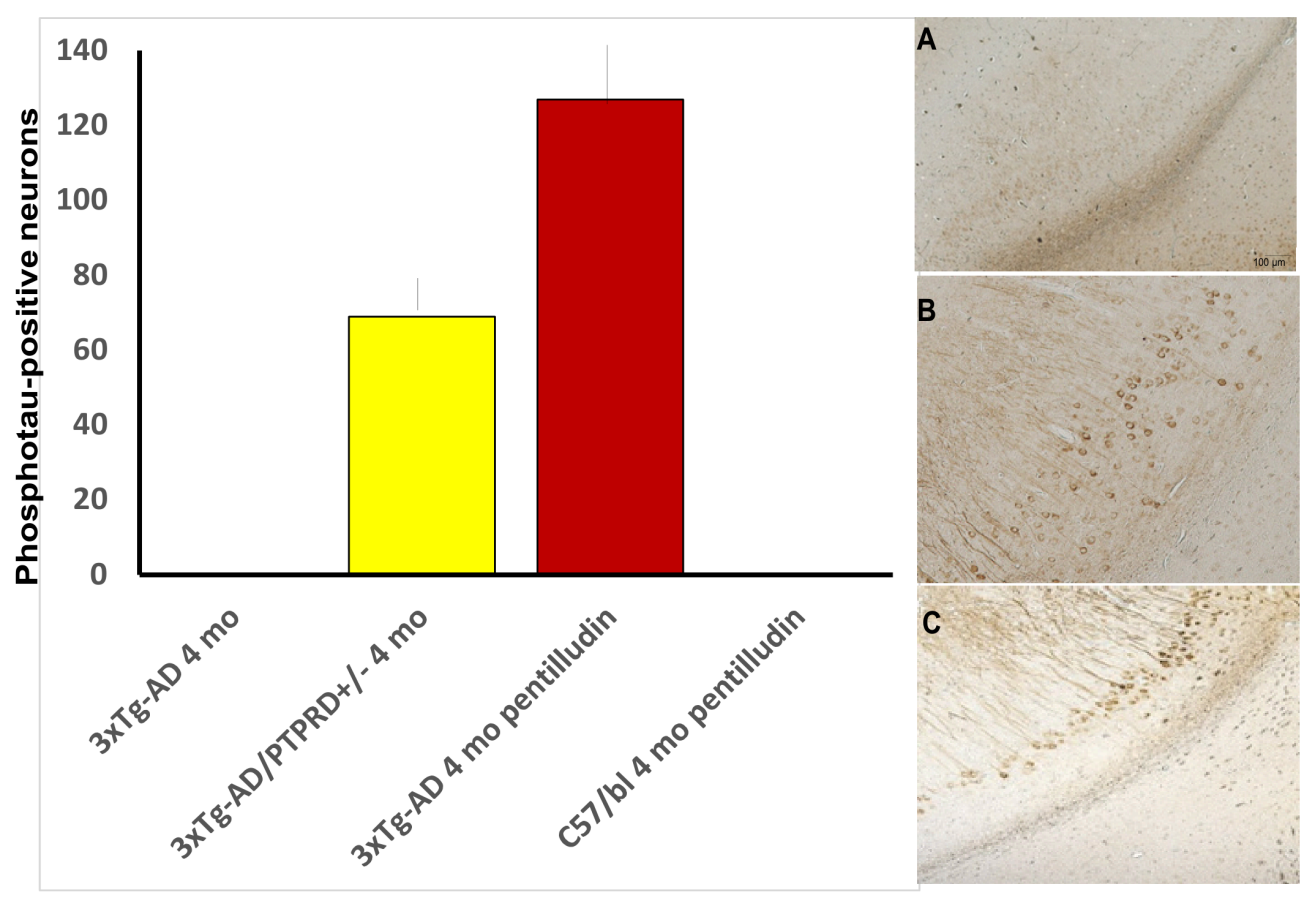
Large effects of genetic or pharmacologic reduction in PTPRD on pathologically phosphorylated tau in 3xTg-AD mice. *Left:* Numbers of phosphotau expressing hippocampal neurons in 4-month-old mice with genetic background and PTPRD inhibitor treatment (pentilludin 25 mg/kg/qod weeks 6–16) as indicated. Numbers are essentially zero in 4-month-old 3xTg-AD mice and in pentilludin-treated wildtype C57bl6 mice. *Right:* photomicrographs of phosphotau/AT-8 immunoreactivity in sections of hippocampus from 4-month-old mice: (**A**) 3xTg-AD, (**B**) 3xTg-AD/PTPRD+/−, Scale = 100um. (**C**) 3xTg-AD treated with pentilludin, Scale = 100um. Brains from wildtype C57bl6 mice treated with pentilludin display results equivalent to those in panel (**A**) (ref. [29]).

Remarkably, 3xTg-AD/PTPRD+/− mice display hyperphosphorylated tau in hippocampal neurons by 4 months of age (Figure 4). PTPRD reduction leads to the appearance of neuronal hippocampal hyperphosphorylated tau that is not only much earlier but also much more robust than the levels found in 3xTg-AD mice with normal, wildtype levels of PTPRD expression [29]. The effects of reduced PTPRD expression on the densities of senile plaque-like pathology identified by its Aβ-immunoreactivity in 20-month-old mice are much more modest, although statistically significant [29].

Pentilludin is a selective PTPRD inhibitor with 690 nM potency and reversible, dose-limiting toxicity in rats noted only at doses > 150 mg/kg/d for two weeks [30]. Administration of pentilludin (100 mg/kg po every other day (qod)) to 3xTg-AD mice from weaning (week 6) to the time of sacrifice at week 16 provides no obvious toxicity. This treatment dramatically increases the number of hippocampal neurons that express pathologically hyperphosphorylated tau (Figure 4) [29]. This increase is even greater than the increase noted when we add heterozygous PTPRD knockout to the 3XTg-AD background. There is no effect of pentilludin in wildtype C57 mice. Conceivably, this result in wildtype animals may support the requirement for the presence of other AD risk alleles for effects of PTPRD activity to be manifest. Such a requirement is consistent with the greater effects of dietary intake of PTPRD-positive allosteric modulator flavonols in humans with ApoE4 AD risk alleles [14,30] (*see below*). These results complement our results from studies of genetic reductions in PTPRD expression. They validate the role of “midlife” alterations in PTPRD activity in contributing to neurofibrillary tau pathology.

**Figure 5:**
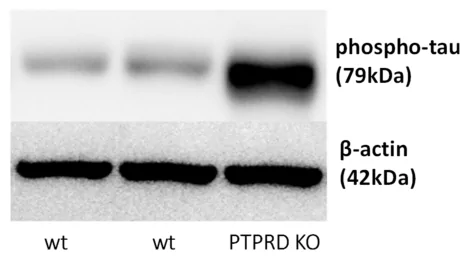
Increased hyperphosphorylated tau in brains of 20-month-old PTPRD knockout mice vs. wildtype (WT) controls. Western analyses of AT-8 immunoreactivity (top bands) and β actin loading control (bottom bands) (*BK, GRU unpublished observations with two wildtype animals shown to allow an assessment of variability, 2024 independently verified in ref.* [31]).

Adding heterozygous knockout to the 3xTg-AD genetic background leads [29] to a trend toward reduced performance on cognitive testing, a possible fit with the modest PTPRD human associations with cognitive functions noted above. By contrast, homozygous PTPRD knockout, studied separately or on the 3xTg-AD genetic background, confers substantial deficits in cognitive abilities, as also noted in a human PTPRD knockout case report [3,20,29]. Homozygous PTPRD knockout also dramatically increases the brain accumulation of hyperphosphorylated tau [31] (Figure 5).

*Strengths and weaknesses:* 3xTg-AD/PTPRD+/− mice provide an AD model that adds to the burden of AD-like neurofibrillary pathology found in 3xTg-AD mice [28,29]. The effects of pharmacological PTPRD inhibition from weaning to 4 months of age in 3xTg-AD mice support these genetic results. The viability of female and male 3xTg-AD/PTPRD+/− mice to 4 months of age and beyond allows for good confidence in the presence of effects in both sexes. Males and females display virtually identical effects of reduced PTPRD expression.

We await replication by others. We are not aware of studies of the effects of reduced PTPRD expression on pathologies that develop in other AD mouse models [32].

*Interpretation of combined human GWAS and 3xTg-AD/PTPRD+/− data:* There is good statistical significance for the effects of reduced PTPRD expression or activity on the development of neuronal neurofibrillary pathology in mice with 3xTg-AD genetic backgrounds. In a Bayesian sense, there were elevated prior probabilities of these results, based on the human associations between 5′ intron PTPRD genomic variants, densities of neurofibrillary pathology in AD brains and expression of PTPRD mRNA. These elevated prior probabilities confer greater confidence in the posterior probabilities for the results obtained in our mouse studies.

Human and mouse model genetics, taken together, now strongly support roles for lifelong “trait” variation in PTPRD in altering the extent of AD neurofibrillary pathophysiology. Pentilludin effects in 3xTg-AD mice indicate that even midlife reductions in PTPRD activity can provide abundant neurofibrillary pathology on this vulnerable 3xTg-AD genetic background.

## 4. Glycogen Synthase Kinase 3s and Their Regulation by Tyrosine Phosphorylation

**Figure 6:**
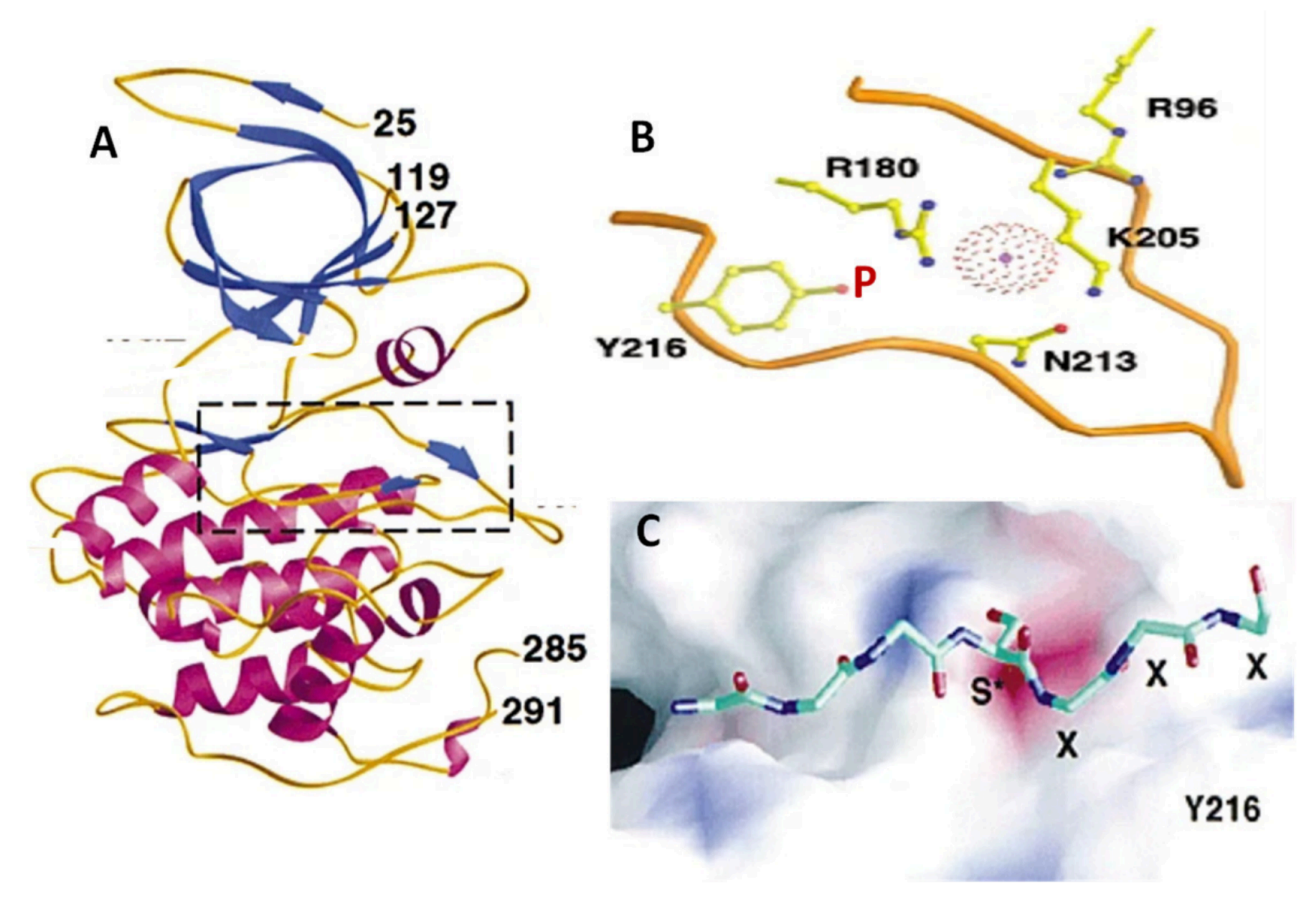
A molecular basis for regulation of GSK3 activity. (**A**) Crystal structure of GSK3β. Box (dashed) overlies the activation domain. (**B**) Activation domain with activating phosphorylated tyrosine 216 (Y216) shown. (**C**) Substrate peptide with serine phosphorylation site (S*) oriented toward the GSK3β catalytic site (red) and nearby Y216 (ref. [34]).

Glycogen synthase kinases α and β are serine/threonine protein kinases that are expressed in neurons (β > α) as well as in a variety of other tissues [33]. Activities of GSKs are robustly modulated by phosphorylation of the tyrosines 216 (GSK3β) and 279 (GSK3α) that lie within the activation domains that lie adjacent to their active catalytic sites [34,35] (Figure 6).

In the brain, the majority of GSK3 tyrosines 216 and 279 are constitutively autophosphorylated [35]. Substantial regulation of the activities of GSK3s could thus come from modulation of the rates of dephosphorylation of these phosphotyrosines by protein tyrosine phosphatases expressed in the same cells.

*Strengths and weaknesses:* There has long been evidence for regulation of the activities of GSK3s by constitutive phosphorylation of the tyrosines that lie in their activation domains [34,36,37]. However, documentation of the large extent of phosphorylation of these regulatory tyrosines in brain (and the modest amount of phosphorylation at alternative putative regulatory sites) is relatively recent [35].

## 5. Glycogen Synthase Kinase 3s Play Key Roles in Tau AD Neurofibrillary Pathophysiology

Hyperphosphorylation plays a key role in tau’s shift from its normal physiological role as an essential contributor to microtubule structure and function to its pathological role in forming the twisted filamentous structures found in neurofibrillary tangles [38,39].

**Figure 7:**
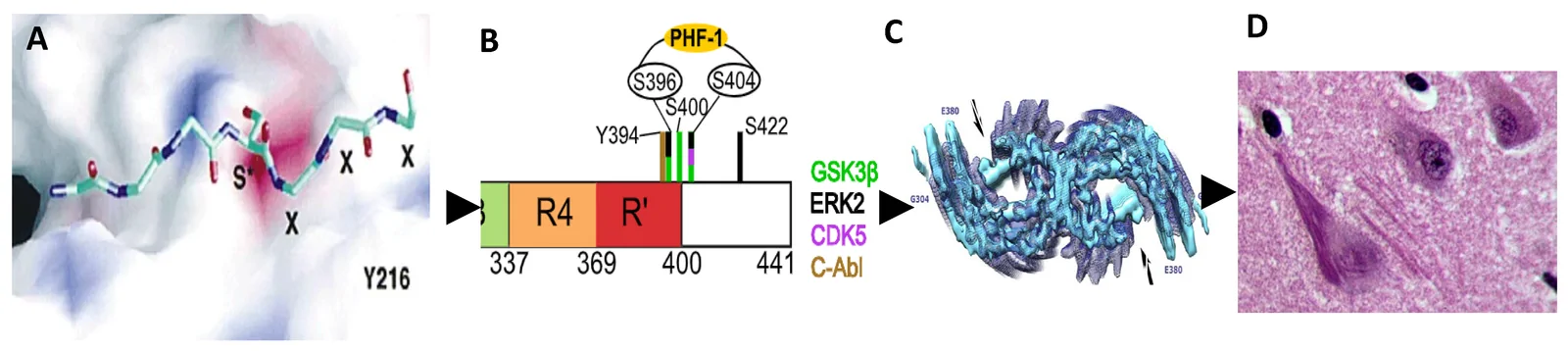
GSK3 (**A**) phosphorylation of serines in tau’s “PHF1” domain (**B**) leads to (**C**) twisted structures in vitro (light blue, cryoEM) that overlap with the structures of extracts from brain (darker blue) that form neurofibrillary pathology (**D**) in Alzheimer’s disease brains. Heights of bars in (**B**) reflect the relative extent of tau phosphorylation in its C-terminal PHF1 domain by the different kinases shown in the same colors (refs. [34,35]).

A variety of evidence suggests that the GSK3s play a central role in this pathological tau hyperphosphorylation. While other serine/threonine kinases can phosphorylate tau, GSK3α and GSK3β are among the most active kinases in phosphorylating tau in vitro [40]. The pattern of tau phosphorylation mediated by GSK3β is also distinctive (Figure 7). GSK3-mediated phosphorylation is focused on the aggregation-associated PHF-1 domain within tau’s C-terminal domain [41] (Figure 7). Tau hyperphosphorylation by GSK3β robustly and virtually uniquely enhances tau aggregation in vitro, leading to formation of tau aggregates with morphologies reminiscent of those found in AD brains [41] (Figure 7).

In vivo evidence also supports roles for GSK3s in AD and/or tau pathophysiology. Lithium inhibits GSK3 activity throughout the body [42]. There are lower rates of AD in humans exposed to lithium via drinking water [43], through treatments for bipolar disorder [44] or in clinical trials (most of which were of modest size, *e.g.*, [45]). In 3xTg-AD mice, lithium depletion increases AD pathology [46]. Small-molecule GSK3 inhibitors [47] also display beneficial effects in mouse models of AD pathology [48].

*Strengths and weaknesses:* GSK3 acts in many ways that have been linked to AD pathophysiology through a variety of routes [49]. For example, GSK3s, especially GSK3β, are also implicated in the pathophysiology that leads to the formation of senile plaques [50]. These effects may complement the effects of GSK3 modulation on neurofibrillary pathophysiology. They also lead to uncertainty about the exact proportion of the cognitive benefits of GSK3 modulation that should be ascribed to GSK3 effects on neurofibrillary pathophysiology.

GSK3s also play many roles in normal physiology [51]. The GSK3s α and β are each expressed in many tissues at levels greater than or equal to those in the brain [40,52]. There is more GSK3α expression in the testis, ovary, lung, kidney and heart than in the brain [53]. GSK3 β is expressed in the brain and kidney at about the same levels, and with only modestly lower levels of expression in the ovary, lung, liver and heart [53]. Modulating GSK3 activity throughout the body via small molecules is thus likely to have consequences that limit doses and thus limit the changes that can be induced in the neurons in which we wish to modulate neurofibrillary pathophysiology.

## 6. PTPRD Dephosphorylates Glycogen Synthase Kinase 3s

Phosphotyrosine GSK3s (pYGSK3s) are now well supported as *bona fide* substrates for PTPRD. Brains of knockout mice with reduced PTPRD expression display increased levels of these pYGSK3s [13]. *C. elegans* with knockouts of their PTPRD homolog also display increased levels of GSK3 phosphoprotein [54].

**Figure 8:**
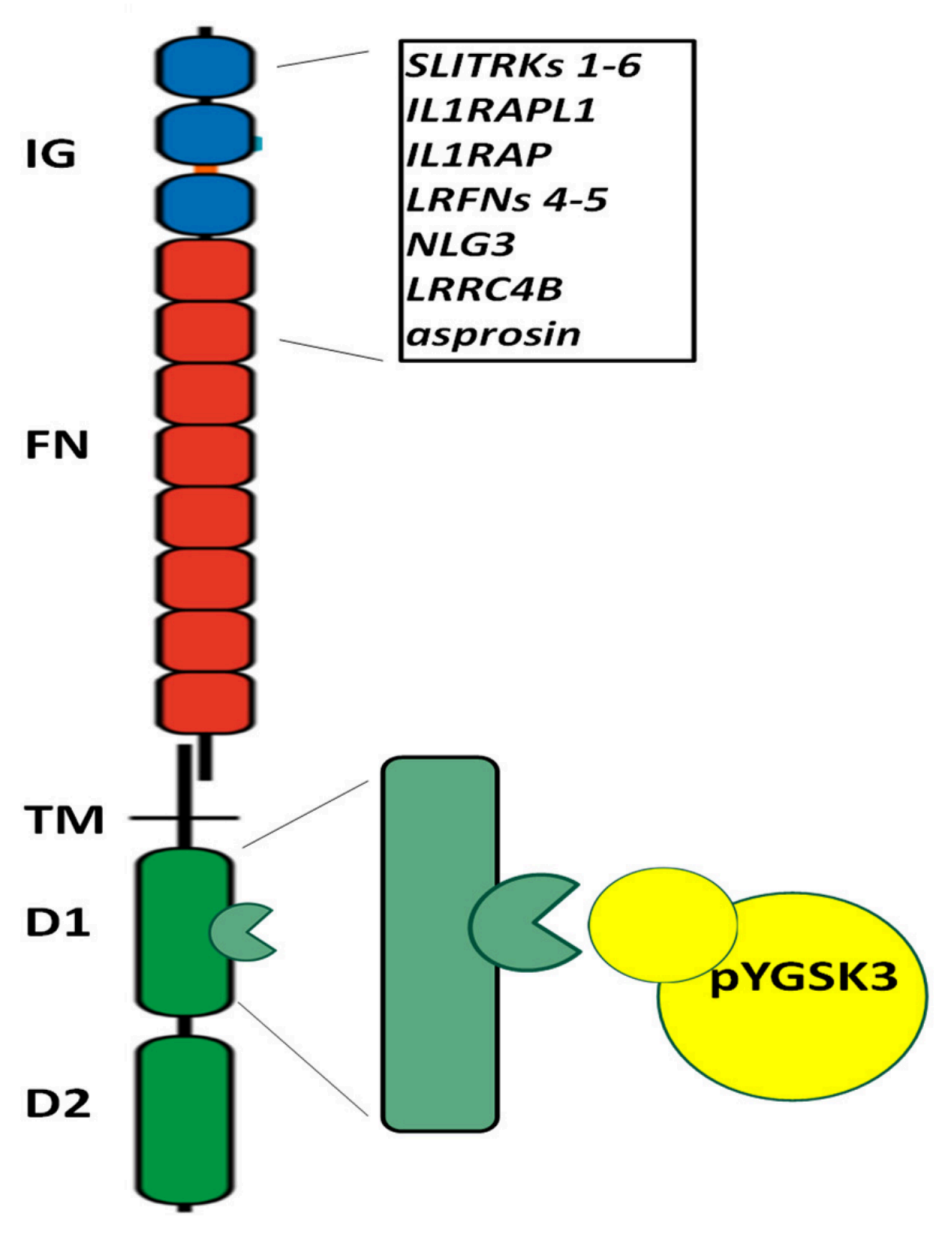
PTPRD schematic showing enlarged D1 phosphatase domain with pYGSK3 as a prime substrate. (Other labels as in Figure 1).

In vitro, PTPRD robustly dephosphorylates a phosphopeptide that contains pY in the middle of the highly homologous sequence that is shared by pY279 GSK3α and pY216 GSK3β [13]. Figure 8 thus adds to Figure 1, positioning pYGSK3 as a prime substrate for PTPRD in coexpressing neurons.

*Implications of PTPRD interactions with glycogen synthase kinase 3s:* As noted above, activities of GSK3α and GSK3β are enhanced by largely constitutive autophosphorylation of their tyrosines 279 and 216 [13,36,54,55]. Within neurons that coexpress these GSKs and PTPRD, GSK3 activities can thus be reduced by activating PTPRD and enhancing dephosphorylation of pY279 GSK3α and pY216 GSK3β.

*Strengths and weaknesses:* As we note above, the substantial evidence for constitutive autophosphorylation of pY279 GSK3α and pY216 GSK3β in the brain [35] strongly suggests that regulating the rates of their dephosphorylation by PTPRD is likely to exert a large impact on the regulation of GSK3’s activities. There is additional complexity possible; however, since GSK3s are also phosphorylated on other tyrosine, serine and threonine residues, including residues whose phosphorylation “primes” phosphorylation at pY279 and pY216 [35].

## 7. PTPRD Is Well Positioned to Alter Neurofibrillary Pathology via GSKs In Vivo with Specificity

PTPRD is largely expressed in neurons [15,16], including cortical and hippocampal neurons in which the most-studied neurofibrillary pathology develops in AD brains. Allen Institute single-cell mRNA-seq data shows that all of the human cerebral cortical/hippocampal cell types that express significant levels of GSK3 β or α mRNAs also express PTPRD mRNA.

Levels of PTPRD expression are also substantially more neuronal than the distribution of GSK3s expression noted above. RNA-seq data shows almost 250 PTPRD tags/million in human cerebral cortex, only 20–25 tags/million in kidney, pituitary and ovary and even fewer in other tissues studied [56].

Data that support the likelihood of direct PTPRD/GSK3 interactions within the same neurons are supplemented by our recent mouse and human results that document PTPRD colocalization with neurofibrillary pathology.

**Figure 9:**
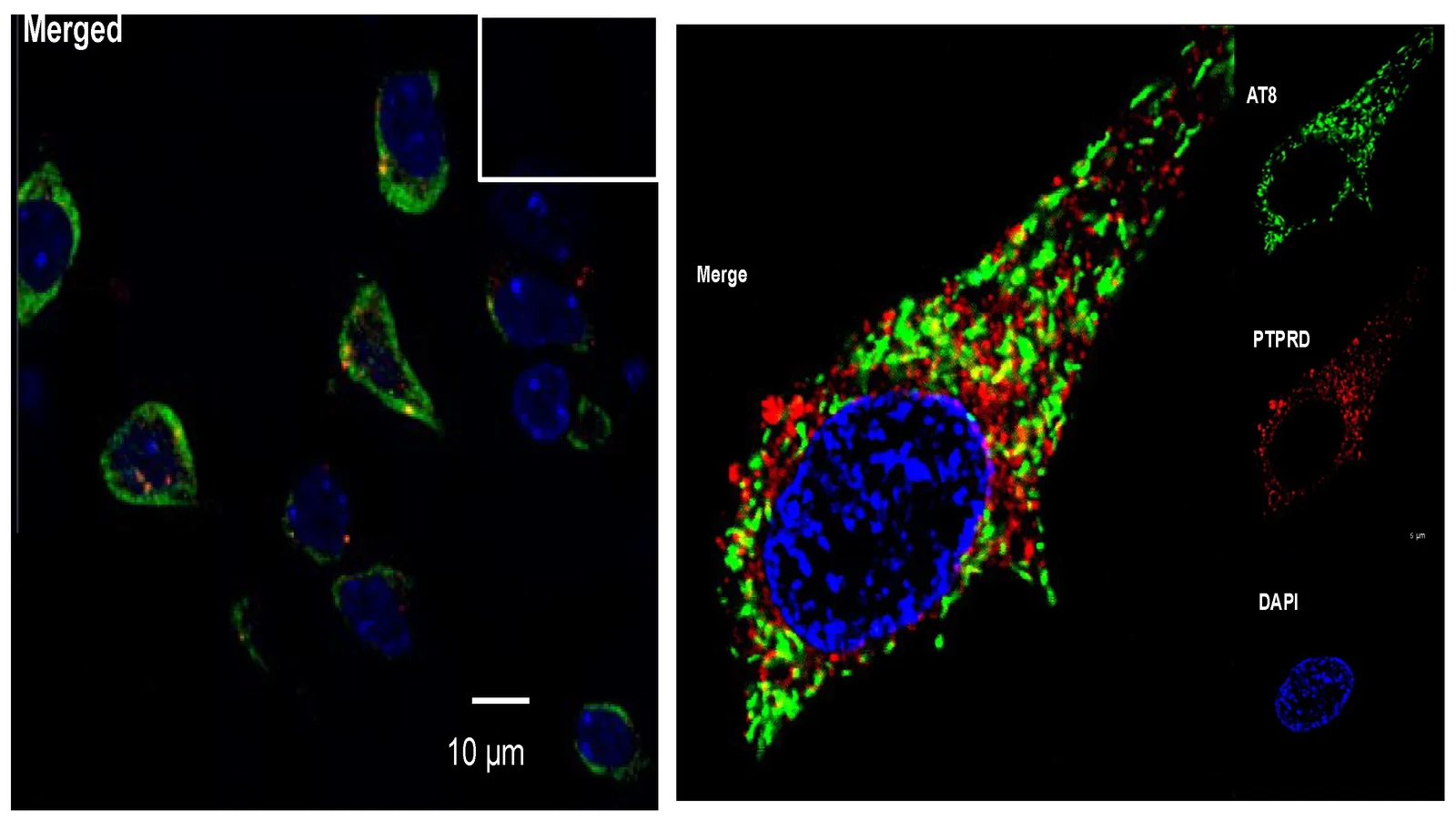
(**Left**) Neurons showing PTPRD (red) and pathologically hyperphosphorylated tau (AT-8, green) immunoreactivities with DAPI nuclear staining (blue) in sections from (top) hippocampus of 3xTg-AD mouse treated with pentilludin. (**Right**) AD cortex. PTPRD staining is absent when antibody is preabsorbed with the cognate PTPRD peptide (white box inset in top image). (*JC, BK, GRU unpublished observations, 2025-6, awaiting independent replication*).

- Many of the hippocampal neurons that display neurofibrillary pathology in pentilludin-treated 3xTg-AD mice also express PTPRD (Figure 9, left panel).
- Many of the neurons from Alzheimer’s disease brains that display pathologically hyperphosphorylated tau also express PTPRD (Figure 9, right panel).

It is worthwhile to re-highlight the comparisons between the cell type and tissue specificity of expression of PTPRD vs. the GSK3s. Outside of the brain, there are large amounts of GSK3 expression vs. comparatively small amounts of PTPRD expression [53,56].

The anatomy of PTPRD/GSK3 co-expression in many of the neurons that are vulnerable to the development of neurofibrillary pathology in humans and mouse models provides a promising substrate for prevention and treatment strategies with enhanced cell type specificity vs. whole-body GSK3 inhibitors.

*Strengths and weaknesses:* Double labeling immuno-histochemistry is possible with high quality antibodies that can recognize specific epitopes in sections from mouse and human brains. PTPRD immunoreactivity is difficult to identify in 3xTg-AD/PTPRD+/− mice (*see below*) and in brains of humans with long postmortem intervals. Nevertheless, we can stain PTPRD successfully in the brains of 3xTg-AD mice treated with pentilludin and in brains from humans with shorter postmortem intervals. There is evidence for specificity: staining is absent when the antibody is preadsorbed by incubation with the cognate PTPRD peptide. The patterns of immunostaining accord well with the Allen brain atlas in situ hybridization results that localize PTPRD mRNA, The specificity of the antiphospho-tau AT-8 antibody has also been extensively validated [57].

The human data sends an important message: upregulating the activity of the PTPRD that continues to be expressed in many of the hippocampal and cortical neurons that display neurofibrillary pathophysiology might be able to curb the continuing development of this pathophysiology and thus alter the course of AD.

## 8. Flavonols Provide Selective Positive Allosteric Modulation of PTPRD Dephosphorylation (Downregulation) of GSK3s in Coexpressing Neurons

**Figure 10:**
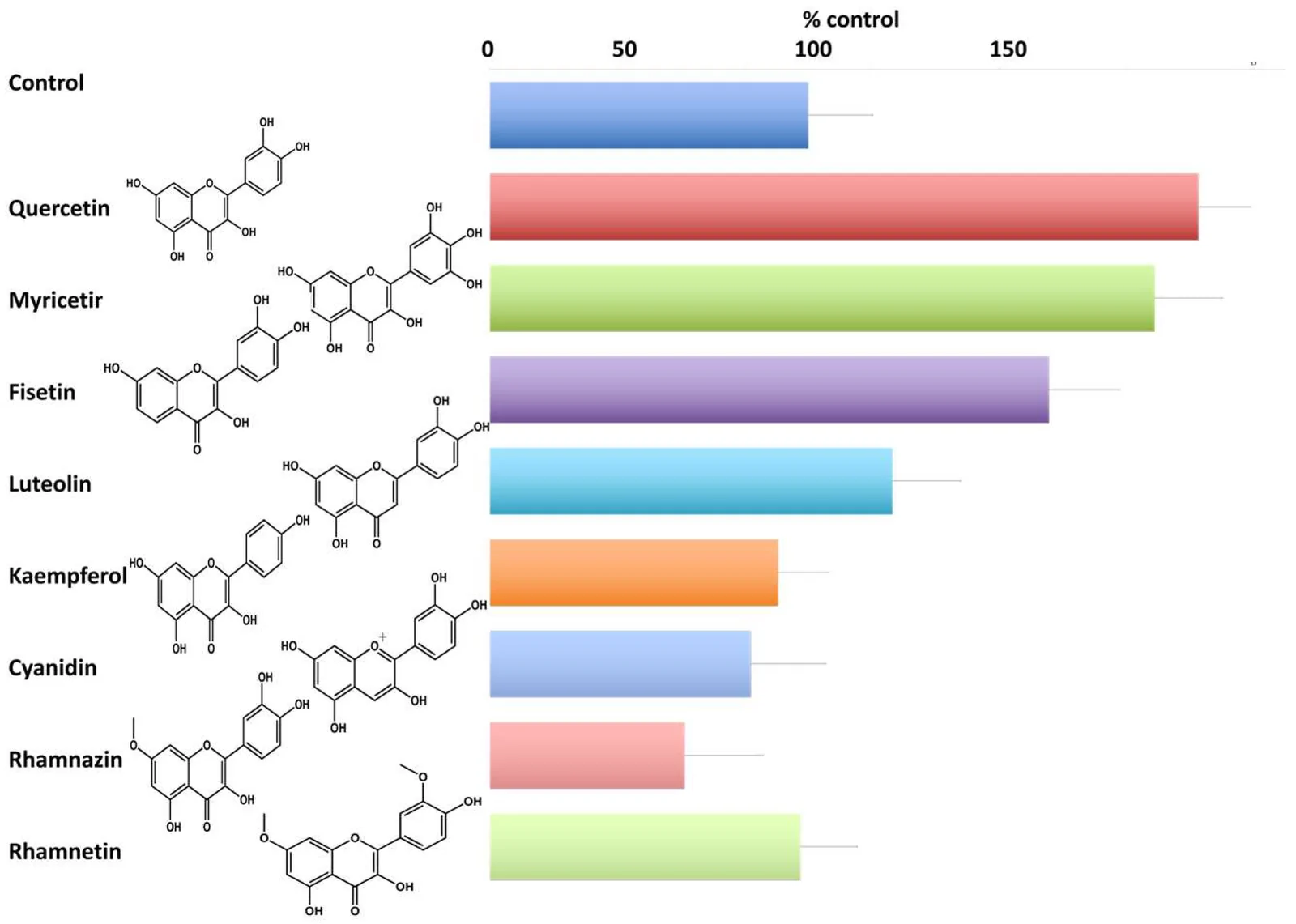
Lead compound PTPRD PAM quercetin enhances PTPRD activity in dephosphorylating pYGSK3. Initial structure–activity relationship provided by compounds whose structures are shown (ref. [13]).

We sought to identify compounds that might be active at PTPRD based on results from our addiction-related GWAS data [58] to which GWAS results in RLS [24] and then in AD [8] were added over time. We followed leads including hints from a screen of small-molecule libraries at the related phosphatase from PTPRS [59]. A flavonoid (scutellarin) inhibited PTPRS activity. We thus screened a group of flavonoids for activity at PTPRD’s phosphatase [13]. To our initial surprise, the abundant dietary flavonols quercetin and myricetin *enhanced* PTPRD dephosphorylation of pYGSK3 (Figure 10). There was evidence for selectivity and specificity. First, quercetin’s positive allosteric modulation (PAM) activity at PTPRD was relatively selective for pYGSK3. PTPRD’s actions in dephosphorylating most of the other top 30 candidate PTPRD substrates were not enhanced by quercetin. Second, quercetin had less PAM effect on pYGSK3′s dephosphorylation by related phosphatases from PTPRS, PTPRF and PTPRJ. Third, there was a striking structure–activity relationship even in our initial studies of natural product flavonoids [13]. Small changes in structure provided large changes in activity. Molecular modeling provided a plausible, though speculative, mechanism for these selectivities [13]. Fourth, quercetin subsequently failed to display any potent activity in a EUROFINS screen of virtually all targets of known drugs, with actions at a modest number of sites only at 10^−5^ M concentrations [60].

## 9. Flavonol Analog Structure–Activity Studies Provide Improved In Vitro PTPRD PAM Activities

**Figure 11:**
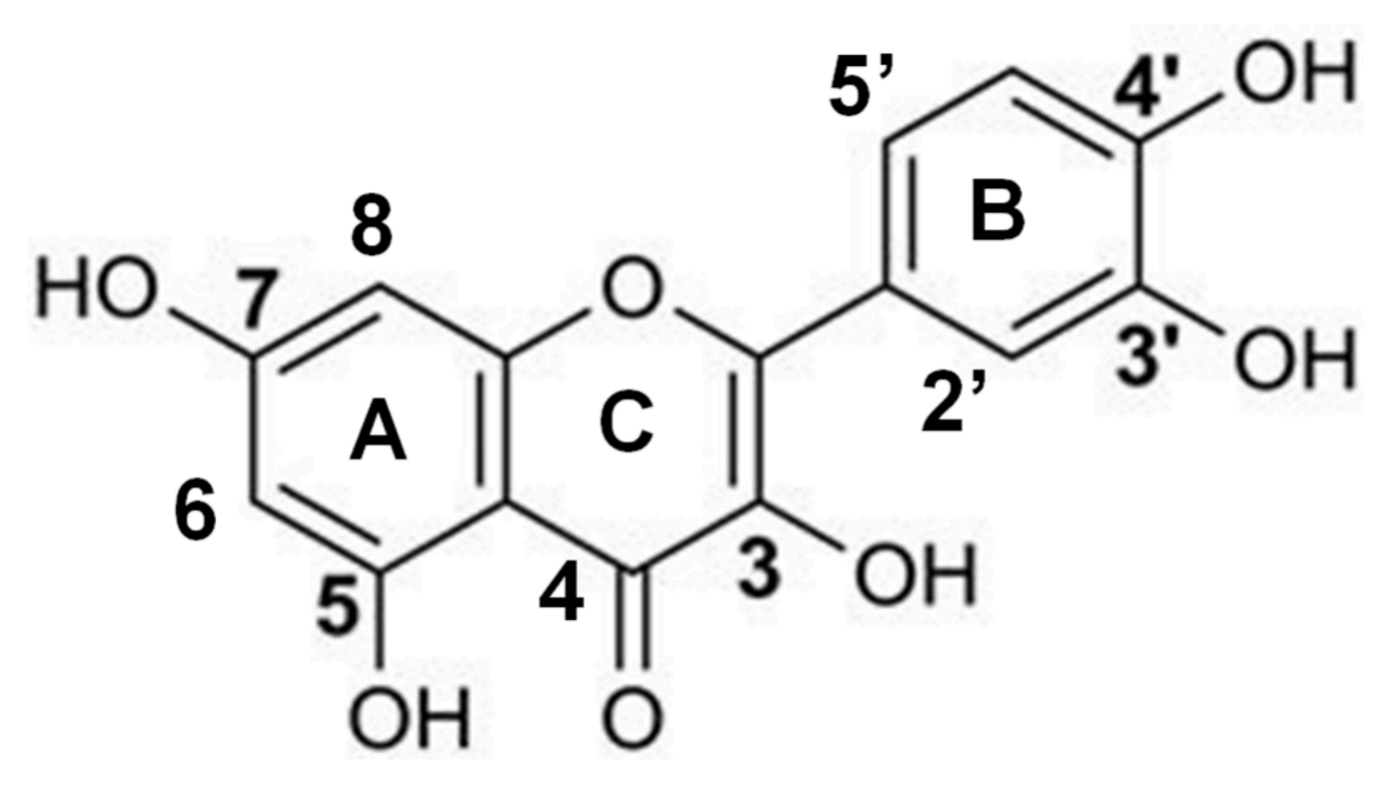
Quercetin structure, position numbers and letters for rings (see https://iupac.qmul.ac.uk/flavonoid/ (accessed 1 April 2026)).

We pursued further structure–activity relationships for PTPRD PAMs using natural product and synthesized quercetin analogs, seeking further evidence for selectivity and working to identify a developmental candidate PTPRD PAM. This work continued to provide substantial evidence for a selective structure–activity relationship underlying PAM activity for PTPRD’s ability to dephosphorylate pyGSK3 (Figure 11 and Figure S1, Tables S1 and S2).

We can reduce quercetin’s PAM activities (e.g., its ability to enhance PTPRD’s pYGSK3 dephosphorylation) by:

- Removing one of its B ring hydroxyls (quercetin vs. kampferol);
- Halogen substitutions for its B ring 3′ hydroxyls;
- Removing its 3-position hydroxyl (vs. luteolin);
- Removing its 4-position keto (vs. cyanidin);
- Removing its 5-position hydroxyl (vs. fisetin);
- Methylating at most positions (vs. rhamnazin and others).

Analogs with changes at other positions retain PTPRD PAM activities with evidence for specificity:

- Myricetin adds a third B-ring hydroxyl group and is almost as active a PTPRD PAM as quercetin.
- There is PAM activity when quercetin’s 8 position is amino-methylated. However, 8-substitution with a variety of other groups reduces PAM activity
- *Excitingly*, *we found full retention of PTPRD PAM activity with 6 bromination*. PAM activities ranged up to 140% of quercetin’s activity in some assays. By contrast, chlorine or iodine 6-substitution provided less PAM activity. 6 O-methylation or hydroxylation eliminated PAM activity. 6,8 dibromoquercetin with halogen substitutions at both the 6 and 8 positions inhibited PTPRD.

The tight structure–activity relationship for 6-position substitutions, and for other substitutions on other parts of the quercetin backbone, provides evidence for the specificity of the interaction.

Early in the process of developing this structure–activity relationship, we conducted in silico molecular docking studies with quercetin and the best available molecular model for PTPRD’s phosphatase [13]. This work identified a favored “PAM” allosteric binding site for quercetin on PTPRD, some quercetin ability to dock at the catalytic site and possible quercetin recognition by two other sites on the phosphatase [13]. Quercetin binding to its favored PAM site selectively altered the docking of pYGSK3 phosphopeptide to the PTPRD phosphatase in silico in ways that appear to explain its PAM activities and fit with observations made from analyses of the structures of cocrystals of other protein tyrosine phosphatases and their substrates [13,61]. However, the structure–activity relationship of 6- and 8-position quercetin analogs does not fit perfectly with this in silico binding site, suggesting the requirements for more refinement to our initial model.

*Strengths and weaknesses:* We have worked to stretch the financial support for the work that was available to our laboratories to provide as full a structure–activity series as possible. Structures of all synthesized compounds were validated by NMR spectra. However, many of the compounds used in screening assays were crystallized but not subject to further extensive purification. All are thus >80% pure, but many are likely <95% pure. Strong candidates, including 6BrQ, are column-purified to >95% purity for the confirmatory studies that include the in vivo work [29] noted below.

In vitro assays for PTPRD PAM activity were performed in triplicate as described [13] for each of the screened compounds on at least two occasions. However, only the most promising 6- and 8-position substituted compounds, as well as quercetin, have been tested in many independent assays, each performed in triplicate, that provide the range of relative PAM activities for, *e.g.*, 6BrQ vs. quercetin that is cited above. It has thus been especially gratifying to note the in vivo 6BrQ activity (*below*) that confirms our in vitro results.

Development of a drug almost always requires intellectual property protection. The Veterans Administration has filed for this (patent) protection for glycosylated 6BrQ analogs to facilitate such subsequent development.

## 10. PTPRD PAMs Reduce Neuronal Accumulation of Hyperphosphorylated Tau in Mouse Models

Quercetin displays effects on development of AD-like pathology in 3xTg-AD and 3xTg-AD/PTPRD+/− mice. Quercetin administered to 3xTg-AD mice during the 3 months (25 mg/kg ip, qod) or 12 months (100 mg/kg po, qod) prior to their sacrifice at ages 18–21 months substantially reduces neurofibrillary pathology [62,63].

**Figure 12:**
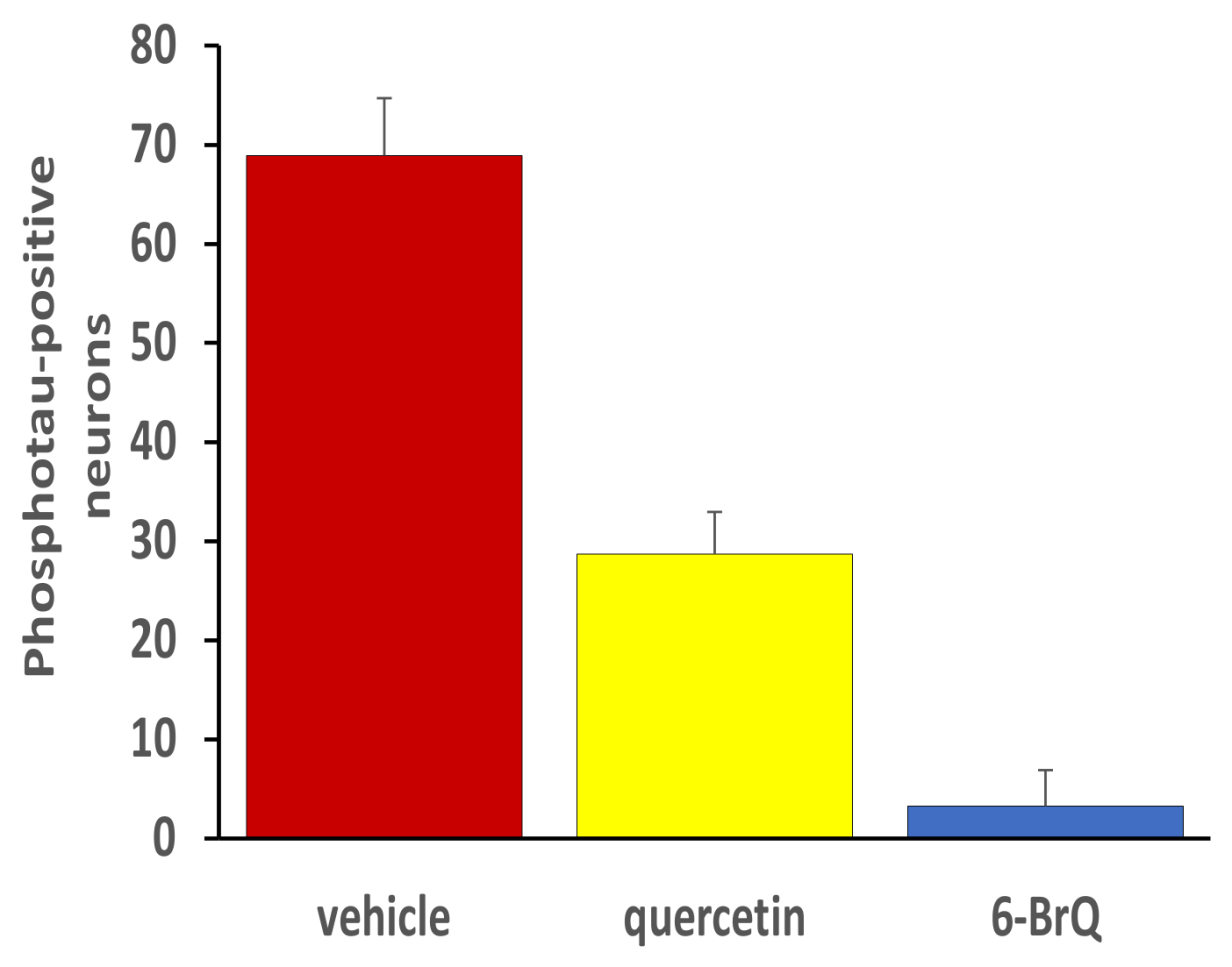
Quercetin and 6BrQ markedly reduce tau pathology in 3xTg-AD/PTPRD+/− brains. Numbers of hippocampal neurons/half-brain section that express hyperphosphorylated tau in 4-month-old 3xTg-AD/PTPRD+/− mice treated from weaning to 4 months of age with vehicle, quercetin or 6BrQ (25 mg/kg, ip, qod). 6BrQ markedly reduces the numbers of hippocampal neurons/mouse half-brain section that display AT8 pathologically phosphorylated tau (ref. [29]).

Quercetin administered intraperitoneally (25 mg/kg, qod from weeks 4 to 16) to 3xTg-AD/PTPRD+/− mice also significantly reduces the development of neurofibrillary pathology in both male and female animals [29] (Figure 12). Mice in these experiments display no signs of specific toxicities [29,62,63].

Our improved PTPRD PAM 6BrQ was well tolerated by 3xTg-AD/PTPRD+/− mice. It was even more active. While vehicle-treated 3xTg-AD/PTPRD+/− mice displayed an average of 69 neurons/half-brain section that expressed hyperphosphorylated tau and quercetin-treated mice displayed 29, mice treated with 6BrQ displayed 3 [29] (Figure 12).

This in vivo work underscores the opportunities that we now have to reduce expression of neurofibrillary pathology, at least in those with genetic predispositions, using bioavailable quercetins and improved quercetin analogs.

### Implications of 6BrQ In Vitro and In Vivo Data for In Vivo Modulation of PTPRD PAM Activities

Our in vitro and in vivo data led us to consider 6BrQ as a candidate for development as an anti-AD compound. However, 6BrQ is likely to share disadvantages that quercetin displays as an oral medication. Quercetin is poorly bioavailable; there are large individual differences in the plasma levels achieved by the same dosing regimens [64,65]. Neither quercetin nor 6BrQ [66] are eligible for composition-of-matter intellectual property protection.

**Figure 13:**
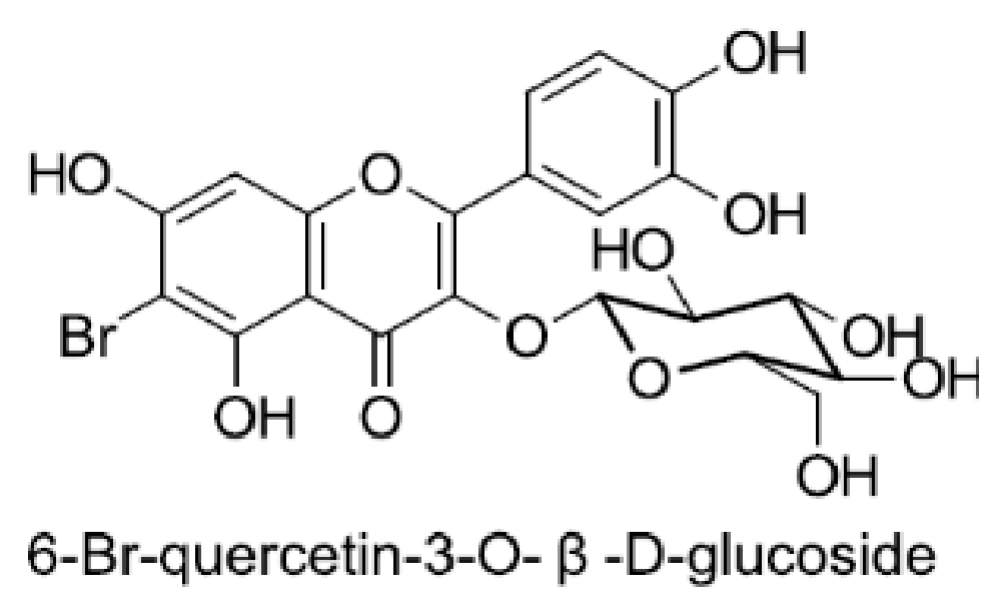
PTPRD PAM drug candidate 6BrQG.

By contrast, there is much-improved oral bioavailability for glycosylated quercetin analogs, which are deglycosylated as they transit from the gut to the bloodstream [64,65,67,68,69]. The 3-O-glycosyl 6BrQG prodrug (6BrQG, Figure 13) is thus (*very*) likely to display the improved oral bioavailability displayed by glycosylated quercetin analogs. This compound or other glycosylated 6BrQ prodrugs allow for the composition-of-matter intellectual property protection that is vital to most drug development.

*Strengths and weaknesses:* The large size of the effects that we identified from quercetin dosing from weaning until 4 months of age in 3xTg-AD/PTPRD+/− mice accords very well with the large effects for parenteral quercetin at the same alternate-day dose that others found on the development of AD-like pathology in 3xTg-AD mice aged to almost 2 years of age [29,63]. Our combined in vitro and in vivo data also accords very well with results of human dietary record studies (*below*) that support sizable effects of flavonol intake on vulnerability of genetically predisposed humans to develop AD.

We have compared the activities of a large number of compounds in vitro. We have only compared quercetin to 6BrQ in vivo. Nevertheless, the remarkably larger benefits of 6BrQ in reducing the accumulation of hyperphosphorylated tau in vivo fit well with our in vitro SAR results. Genetically vulnerable mice and (*below*) humans appear to receive substantial benefits from flavonol administration. These benefits are likely to be enhanced with improved flavonol analogs in humans, as they are in 3xTg-AD/PTPRD+/− mice.

## 11. Humans with Higher Levels of Dietary Flavonol Intake Display Reduced Incidence of Alzheimer’s Disease/ADRD, Especially in Those at Elevated Genetic Risk

**Figure 14:**
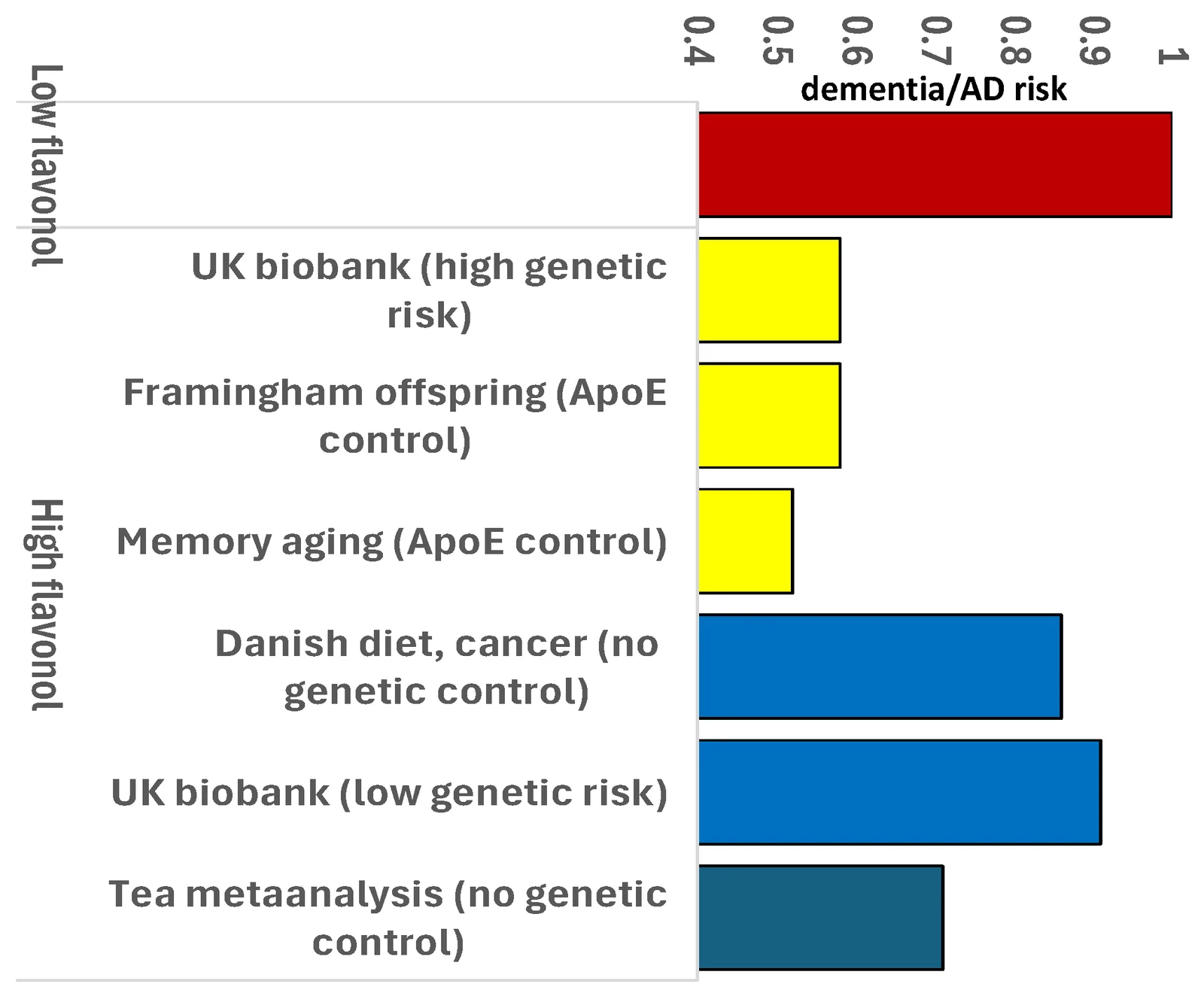
Reduced risk of developing dementia or AD in participants with higher vs. lower intake of flavonols. There are larger effects in individuals at higher genetic risk; smaller effects in individuals at low genetic risk or in studies that did not control for genetic risk (ref. [14]).

As we have recently reviewed [14], results of human dietary record studies provide striking and strong parallels with the effects of quercetin in genetically vulnerable mouse models (Figure 14). These data come from cohorts of individuals who provided estimates of their daily intake of different classes of flavonoids via dietary records and whose ApoE genotypes are known [70,71,72]. During average follow-up periods that range from 9 to 26 years, individuals at higher genetic risk who reported higher flavonol intake displayed striking >40% reductions in the incidence of AD or AD-related dementias compared with those with low flavonol intake [14] (Figure 14). Studies of individuals who lacked common AD risk alleles, or studies that did not control for these alleles, found smaller effects [72,73].

Intake of tea provided the largest source of flavonols, largely glycosylated, in several of these dietary record studies [14]. A meta-analysis of studies of individuals who reported substantial tea drinking also found a significantly reduced incidence of dementia, although there was no control for AD risk genotypes [74] (Figure 14).

Reported intake of flavanones, the flavonoids that are largely derived from citrus and that differ from flavonols based only in the lack of their 3-position hydroxyls, provides no significant association with dementia incidence in any of three samples in which this is reported [14,71,72,73]. This striking difference provides a structure–activity relationship that we can use (*below*) to compare the differential activities of flavanones and flavonols in eliciting other biochemical effects that have been putatively linked to AD.

*Strengths and weaknesses:* A Meta-analysis of studies that combined ginkgo (which contains glycosylated flavonols [64,75]) with cholinesterase inhibitors found significant effects in reducing age-related declines on several cognitive scales [76]. However, late-life supplementation with ginkgo failed to reduce progression to AD in individuals who were not ApoE genotyped [77,78]. It is conceivable that quercetin or 6BrQ/6BrQG will need to be administered earlier in [70,71,72] life to prevent the development of AD in genetically vulnerable individuals.

Diets tend to be stable during much of the lifespan [79]. It is thus difficult to elucidate the effects of the timing of flavonol effects on neurofibrillary pathophysiology from human dietary record studies alone. Studies of the timing of flavonol benefits in mouse models can provide data that can aid the design of subsequent targeted human studies.

There are concerns that high flavonol intake typically correlates with broader diet quality and lifestyle factors that could independently reduce dementia risk and provide a standard limitation of dietary record cohort studies. Covariates applied in the primary analyses of several of these studies sought to correct for this limitation [70,71,72]. Of course, this limitation would be less likely to identify the class selectivities among flavonoids that are identified by the results that we have reviewed in [14].

There may be a signal concerning protection from the development of AD or dementia by anthocyanins. Odds ratios of AD/dementia incidence for highest intake vs. lowest intake groups are 0.78, 1.0, 0.29 and 0.69, for the UK (high genetic risk) [72], Danish [73], Framingham [71] and Memory and Aging [80] samples, respectively. Anthocyanins (glycosylated forms) and anthocyanidins (aglycones) in the US diet come prominently from strawberries, blueberries and black beans [81]. Several tested deglycosylated anthocyanins (anthocyanadins, Table S3 and Figure 12) fail to increase PTPRD’s ability to dephosphorylate pYGSK3 phosphopeptide, however [13]. Anthocyanins are thus good candidates for future research to elucidate mechanisms for contributions of these compounds or of their metabolites.

### Interpretation of Combined Human Dietary Record and Mouse Flavonol/Flavonol Analog Treatment Data

There is strong convergence between the human and mouse model datasets and between these data and the in vitro results noted above. In both in vivo datasets, specific AD-relevant genetic vulnerabilities interact with effects of flavonol consumption or treatment. Human data (flavonols vs. other classes of flavonoids) and mouse model data (quercetin vs. 6BrQ) fit well with in vitro results obtained from studies of flavonoid effects on PTPRD’s phosphatase actions in dephosphorylating pYGSK3 [13,29]. The most parsimonious explanation for these parallels: there are common in vivo mechanisms involved in the flavonol/flavonol analogs’ actions in reducing the development of AD pathology in genetically vulnerable humans and mouse models.

## 12. Molecular and Cellular Bases for “Common In Vivo Mechanism(S) Involved in Flavonol/Flavonol Analogs’ Actions in Reducing Development of AD Neurofibrillary Pathology in Genetically- Vulnerable Humans and Mouse Models”

We have found no high-potency quercetin (or 6BrQ) effects and only a few low-potency effects in the EUROFINS screen at targets for virtually all of the targets of currently used drugs [60]. We can thus evaluate evidence for PTPRD-based mechanisms for these actions of flavonols and flavonol analogs vs. some of the other mechanisms proposed in the literature for flavonol benefits.

Antioxidant effects of quercetin and other flavonols are the most cited in the literature [60]. We can thus compare the antioxidant vs. PTPRD properties of flavonols vs. related compounds to assess the fit with in vivo results.

**Figure 15:**
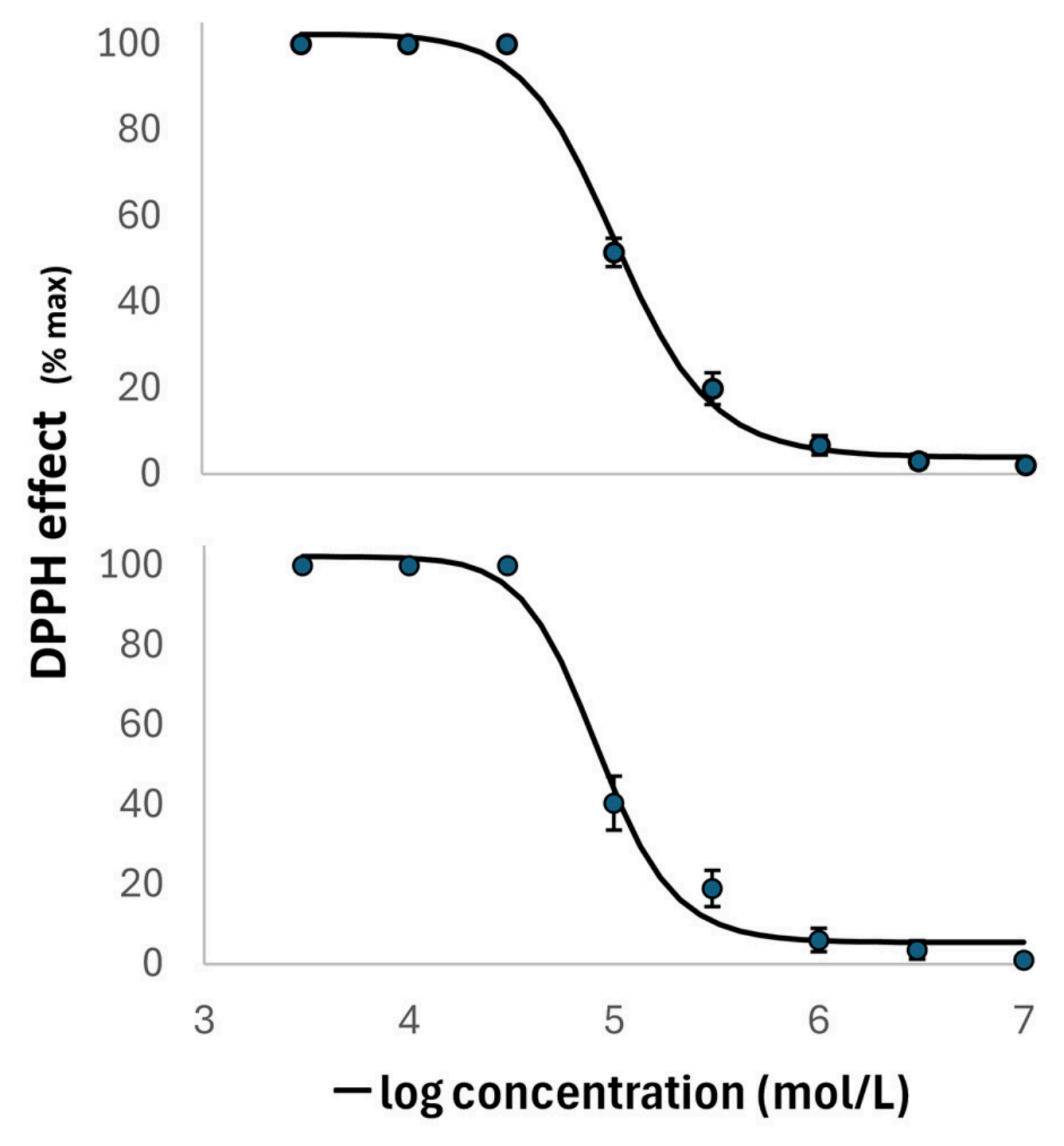
Quercetin (top panel) and 6BrQ (bottom panel) antioxidant effects in DPPH scavenging assays. Calculated EC50 values 10^−5^ and 10^−4.9^, respectively (*KS and E Hess, unpublished observations 2025, we await independent replication*).

- 6BrQ is superior to quercetin as an in vitro PTPRD PAM, superior to quercetin in reducing the accumulation of hyperphosphorylated tau in hippocampal neurons in vivo, but equivalent to quercetin in cyclic voltammetric [60] and in biochemical assays of antioxidant properties (Figure 15).
- Flavanones, whose intake provides no significant association with dementia incidence [14,71,72,73], provide in vitro antioxidant properties similar to those of quercetin, whose intake provides a strong association with dementia incidence [82].

Flavonol effects are also reported at other targets, including neuroinflammation, Aβ aggregation, β secretase activity, acetylcholinesterase, NF-κB, NO, iNOS, TNF, IFN, IL1, IL6, AMPK, Casp3 and 7, COX2, Nrf-2, JUK, PI3K, IL12, as well as Park 2, 5 and 7 [14]. However, none of these reports provides sufficient structure-function data to comprehensively assess their fits with in vivo results.

*Strengths and weaknesses:* Substantial PTPRD contributions to flavonol benefits for the prevention of AD and AD-like neuronal neuropathology in humans and mice with elevated genetic risks are supported by the convergence of existing data. Contributions of PTPRD effects, of course, do not exclude possible contributions of antioxidant and other mechanisms whose effects might be more modest and thus be missed in studies that categorize the effects of different dietary flavonoid components. Further, we lack data that could help to include or exclude involvement of a number of other flavonoid targets that have been postulated in the literature.

## 13. Summary of Evidence Supporting Our Working Hypotheses

**Figure 16:**
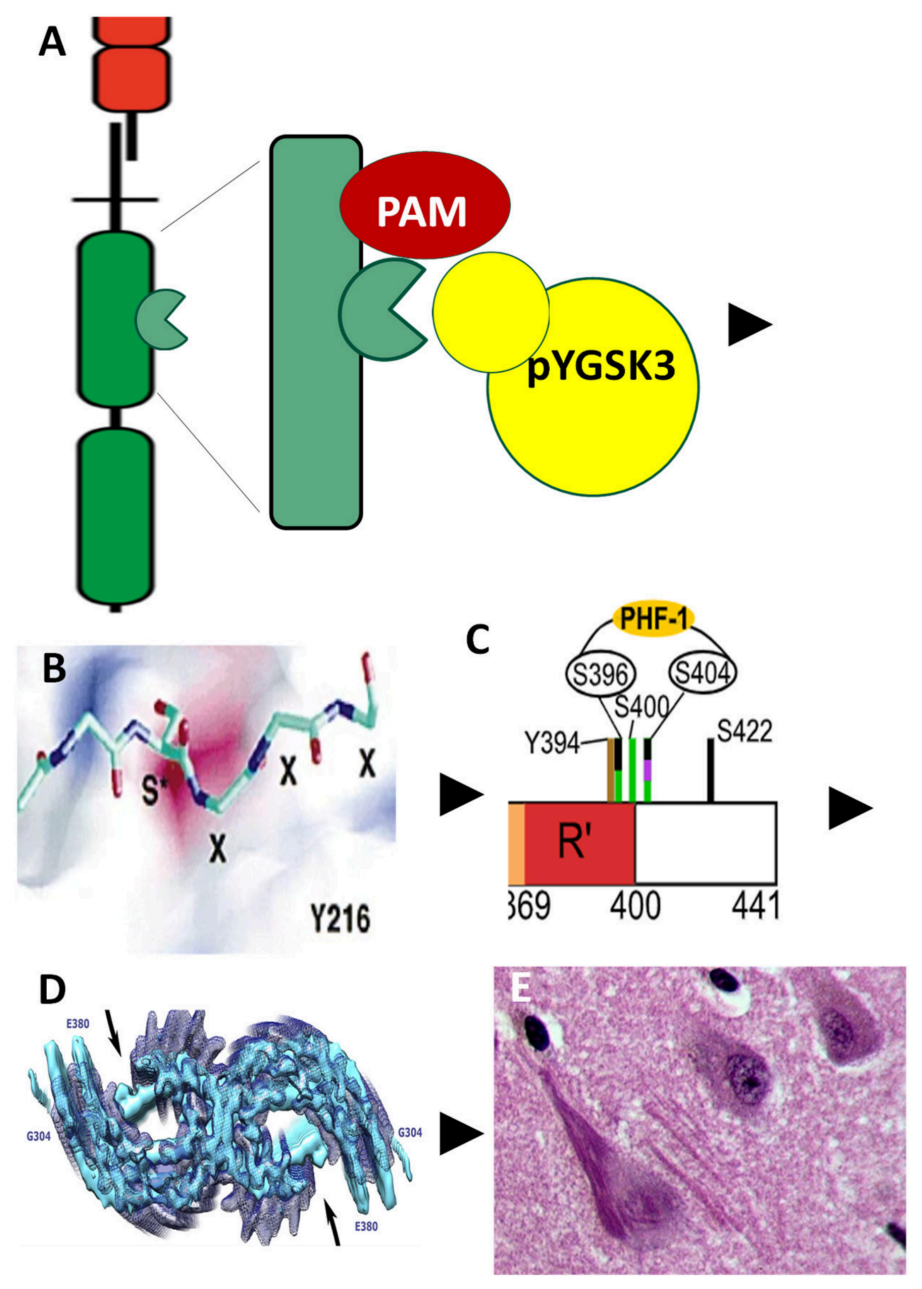
Hypothesis: PTPRD PAMs (**A**) increase activity of PTPRD. More active PTPRD dephosphorylates and downregulates GSK3s in coexpressing neurons (**B**) There is less tau hyper-phosphorylation (**C**), less pathological neurofibrillary pathophysiology (**D**) and less neurofibrillary pathology (**E**).

A convergence of evidence from a variety of approaches now supports our hypotheses [13] that (a) PTPRD plays a key role in the pathogenesis of AD neuronal neurofibrillary neuropathology by making substantial contributions to tau hyperphosphorylation through mechanisms that prominently include its regulation of the activities of GSK3 α and β and that (b) flavonols and improved flavonol analog PTPRD PAMs provide a novel path to possibly preventing and treating AD, especially in those at higher genetic risk (Figure 16).

Supporting data from *humans* include the selective GWAS associations between PTPRD variants, levels of PTPRD expression and densities of neurofibrillary pathology in Alzheimer’s disease brains. They include the associations between flavonol intake and risk of developing AD. Colocalization of PTPRD and neurofibrillary pathology in neurons from AD brains adds to this evidence.

Data from *mouse models* includes the greater neurofibrillary pathophysiology in 3xTg-AD mice with genetic or pharmacologic inhibition of PTPRD. It includes the colocalization of PTPRD in many neurons that express neurofibrillary pathophysiology in 3xTg-AD mice treated with pentilludin. It includes the reductions in neuronal accumulation of neurofibrillary pathology in 3xTg-AD/PTPRD +/− mice treated with PTPRD-positive allosteric modulators quercetin and 6BrQ. Brains of mice with reduced PTPRD expression display more phosphorylated GSK3s pYGSK216 and 279 and more phosphotau in Western blot experiments, supporting GSK3 and phospho tau regulation by PTPRD.

In vitro evidence includes our initial observation of positive allosteric modulation of the activity of PTPRD’s phosphatase by quercetin, the structure–activity relationship that this work with more than 110 natural product and synthetic flavonoids and analogs provided and the substantial evidence for its specificity. When results of dietary record studies documenting flavonols’ specific abilities to reduce the incidence of AD in genetically predisposed individuals appeared, they underscored the remarkable parallels with our in vitro PTPRD PAM activities. In contrast, no other in vitro quercetin activity, including those tested in broad screens, provided such structure–activity relationship parallels with in vivo data.

There remain open questions, including many that we have noted throughout this review. Reviewers of this manuscript have cited the possibility that we have unconsciously selected favorable data to include, the fact that much of the work comes from our research program, the fact that mouse work centers on 3xTg-AD and 3xTg-AD/PTPRD +/− mice and few other mouse strains, the uncertain generalizability across AD models and humans, the validity of observational dietary evidence, the modest bioavailability of quercetin, the limitation of clinical efficacy/target engagement to dietary record studies, the inclusion of data that has not been independently replicated and the appearance of conflict whenever intellectual property protection that is essential to drug development is included in the discussion. Reviewers also cited the genuine strengths of this work, including “triangulation” across methods, a testable pathway, substrate-selective biochemical data, and an emerging structure–activity relationship. We concur that the growing body of evidence from so many sources continues to strongly support our hypotheses concerning PTPRD- and flavonol-modified contributions to AD neurofibrillary pathology.

## 14. Implications for Prevention and Treatment

**Figure 17:**
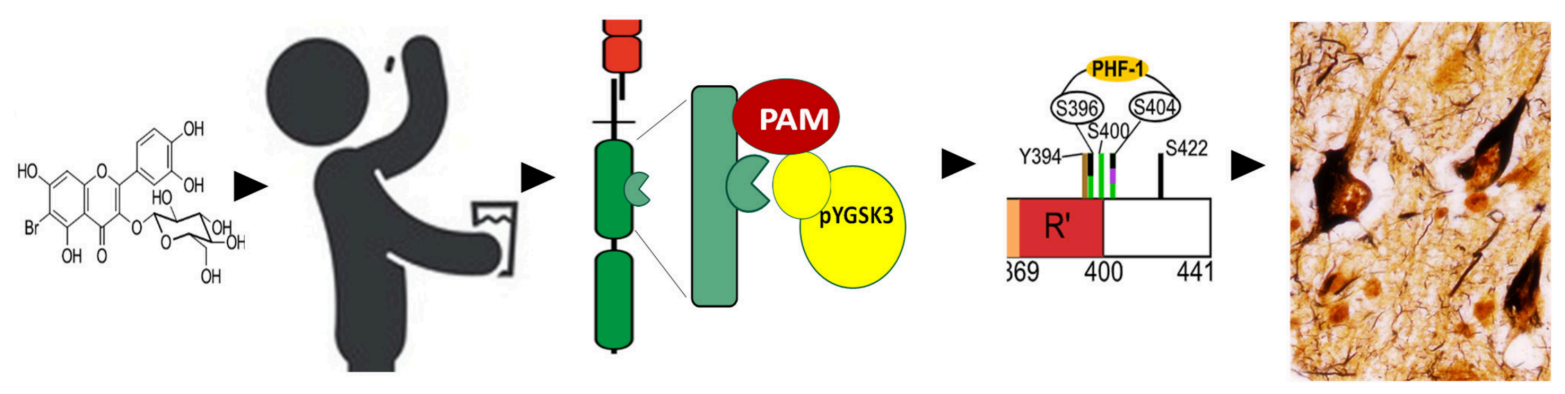
Likely therapeutic implications. Orally bioavailable PTPRD PAM increases PTPRD dephosphorylation of GSK3s which reduces tau hyperphosphorylation and AD neurofibrillary pathophysiology.

The strength of the evidence that supports our hypotheses and the often-devastating consequences of AD neurodegeneration underscore the possibilities and risks involved in using this information to improve AD prevention and treatment, especially in genetically vulnerable individuals (Figure 17).

We are taking a standard pharmacological and pharmaceutical approach to the development of 6BrQG/6BrQ. This involves seeking support and collaborators for several years of preclinical studies that document safety, phase I studies that document safety in normal human research volunteers, phase II studies that document safety in aging individuals, including those with mild cognitive impairment or early Alzheimer’s disease, and phase III studies that document efficacy in slowing the progression of AD and/or in reducing progression from neurologically normal to development of AD. This clinical development will require more than a decade.

Enzymatically modified isoquercetin (EMIQ) is an orally bioavailable glycosylated quercetin analog that has received a generally recognized as safe designation (GRN000220) from the US Food and Drug Administration [83]. Without a prospective clinical trial carried out over many years, it may be premature to suggest that flavonol analogs or EMIQ are now appropriate for use in genetically vulnerable individuals. Nevertheless, the low apparent risks of GRAS EMIQ make it a tempting candidate for use by vulnerable individuals who seek to reduce their risks of developing AD. Individuals with at least one ApoE4 allele and those with strong family histories of AD may be the most likely to seek such possible protection, especially when they compare risks to the elevated risks for developing amyloid-related imaging abnormality (“ARIA”) side effects if they develop AD and use an anti-amyloid antibody AD disease-modifying therapeutic [4,5].

Modest EMIQ doses, currently widely available as dietary supplements, could be tried. The differences in estimated dietary flavonol intake between individuals at higher vs. lower risk for developing AD are modest [14]. However, the incidence of AD, while strikingly lower, was not reduced to zero in the group of genetically vulnerable individuals who reported the highest levels of dietary flavonol intake. Conceivably, EMIQ supplements that provided higher flavonol levels than those provided by even the best diets might reduce AD incidence below even the reduced rates found in individuals with “normal” high flavonol diets.

There is no clear information about the timing and duration of such a dietary supplementation approach from human studies. Late-life ginkgo supplementation has provided mixed results in individuals not selected for AD risk genotypes [14]. Earlier and/or longer flavonol use might be more effective.

The ability to prevent the development of AD pathologies using PTPRD- and flavonol-based approaches are supported by current human and mouse model data. There is less data concerning flavonol efficacies in altering the course of established AD pathophysiology. AD brains do display substantial PTPRD expression in many tangle-bearing neurons. PTPRD PAMs could thus act in such neurons to slow tau pathophysiology and disease progression even in individuals with established AD diagnoses. Clinical trials will need to address the efficacy of flavonol- and PTPRD-dependent mechanisms in slowing ongoing AD pathophysiology and the effects of any such slowing on mnemonic and other AD deficits.

Our recent observations suggest that improved quercetin analogs could exert even greater benefits than EMIQ (Figure 17). As we develop improved evidence for oral bioavailability and safety of these compounds, we are excited about the prospect of providing enhanced benefits in preventing AD in vulnerable individuals and in improving treatments for this devastating disorder.

## Data Availability

No new data were created or analyzed in this study. Data sharing is not applicable to this article.

## Supplementary Materials

The following supporting information can be downloaded at: https://www.mdpi.com/article/10.3390/biomedicines14102210/s1, Figure S1: Key for structures in Tables S1 and S2; Table S1: Quercetin-related structures tested for possible PTPRD PAM activities; Table S2: Anthocyanins studied for possible PTPRD PAM activities. Additional non-anthocyanin structures tested but not shown: eriodictyol, hesperetin, (+)-catechin, (-)-catechin, (-)-epicatechin, gallocatechin gallate and epigallocatechin gallate.

## Abbreviations

AD	Alzheimer’s disease
ApoE	apolipoprotein E
ARIA	amyloid-related imaging abnormalities
6BrQ	6 bromo quercetin
6BrQG	glycosylated 6BrQ prodrug, likely 6 bromo quercetin 3-O-β-D-glucoside
EMIQ	enzymatically modified isoquercetin
GRAS	generally recognized as safe
GSK	glycogen synthase kinase
GWAS	genome wide association
IL1RAP	interleukin 1 receptor associated protein
IL1RAPL1	interleukin 1 receptor associated protein like 1
Ip	intraperitoneal
LRFN	leucine repeat and fibronectin domain
LRR	leucine repeat rich
mRNA	messenger RNA
NFT	Neurofibrillary tangles
NGL1	netrin G ligand 1
PHF1	paired helical filament 1 domain
PTPRD	Receptor type protein tyrosine phosphatase D
pY	phosphotyrosine
qod	every other day
RNA	ribonucleic acid
SNP	single nucleotide polymorphism
Y	tyrosine
