# Supplementary material for: Alzheimer’s Disease Neurofibrillary Pathophysiology: PTPRD- and Flavonol-Modified Mechanisms, Prevention and Therapeutics
Source: Biomedicines. 2026 Sep 29;14(10):2210. doi: 10.3390/biomedicines14102210 (PMC13701322; doi:10.3390/biomedicines14102210)
Supplement: Supplementary file 1 [file biomedicines-14-02210-s001.zip › biomedicines-4477462-supplementary.pdf]

Supplementary Material:

Figure S1: Key for structures in Tables S1 and S2

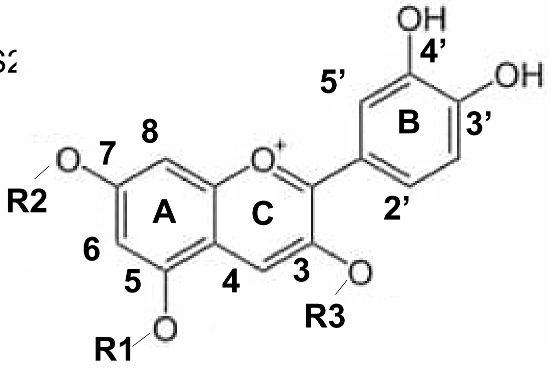

Table S1: Quercetin-related structures tested for possible PTPRD PAM activities

5	6	7	8	3’	2’	4’	3	5’	common name
-OH	-H	-OH	-CH_2NH_2	-OH	-OH	-H	-OH	-H	
-OH	-H	-OH	-CH_2NHOH	-OH	-OH	-H	-OH	-H	
-OH	-H	-OH	-CH_2NHCH_3	-OH	-OH	-H	-OH	-H	
-OH	-H	-OH	-$CH_2NHCH_2CH_3$	-OH	-OH	-H	-OH	-H	
-OH	-H	-OH	-$CH_2NHCH_2CH_2CH_3$	-OH	-OH	-H	-OH	-H	
-OH	-H	-OH	-$CH_2NHCH_2CH_2CH_2CH_3$	-OH	-OH	-H	-OH	-H	
-OH	-H	-OH	-$CH_2NHCH_2CH_2CH_2CH_2CH_3$	-OH	-OH	-H	-OH	-H	
-OH	-H	-OH	-$CH_2NHCH_2CH_2NH_2$	-OH	-OH	-H	-OH	-H	
-OH	-H	-OH	-$CH_2NHCH_2CH_2NHAc$	-OH	-OH	-H	-OH	-H	
-OH	-H	-OH	-$CH_2NHCH_2CH{=}CH_2$	-OH	-OH	-H	-OH	-H	
-OH	-H	-OH	-CHO	-OH	-OH	-H	-OH	-H	
-OH	-H	-OH	-CH_2NH(*i*-prop)	-OH	-OH	-H	-OH	-H	
-OH	-H	-OH	-$CH_2N(CH_3)_2$	-OH	-OH	-H	-OH	-H	
-OH	-H	-OH	-$CH_2N(CH_3)CH_2CH_3$	-OH	-OH	-H	-OH	-H	
-OH	-H	-OH	-$CH_2N(CH_3)CH_2CH_2CH_3$	-OH	-OH	-H	-OH	-H	
-OH	-H	-OH	-$CH_2N(CH_3)CH_2CH_2CH_2CH_3$	-OH	-OH	-H	-OH	-H	
-OH	-H	-OH	-CH_2NHCH_2-CCH	-OH	-OH	-H	-OH	-H	
-OH	-H	-OH	-$CH_2NHCH_2CH_2OH$	-OH	-OH	-H	-OH	-H	
-OH	-H	-OH	-$CH_2NHCH_2CH_2OCH_2CH_2OH$	-OH	-OH	-H	-OH	-H	
-OH	-H	-OH	-CH_2NHCH_2COOH	-OH	-OH	-H	-OH	-H	
-OH	-H	-OH	-$CH_2NHCH_2CH_2NH_2$	-OH	-OH	-H	-OH	-H	
-OH	-H	-OH	-$CH_2NHC(CH_2OH)_3$	-OH	-OH	-H	-OH	-H	
-OH	-H	-OH	-CH_2NH-(isobutyl)	-OH	-OH	-H	-OH	-H	
-OH	-$CH_2N(CH_3)_2$	-OH	-$CH_2N(CH_3)_2$	-OH	-OH	-H	-OH	-H	
-OCH_3	-H	-OCH_3	-H	-OH	-Cl	-H	-OH	-H	
-OH	-H	-OH	-H	-OH	-Cl	-H	-OH	-H	
-OCH_3	-H	-OCH_3	-H	-Cl	-OH	-H	-OH	-H	
-OH	-H	-OH	-H	-Cl	-OH	-H	-OH	-H	
-OCH_3	-H	-OCH_3	-H	-OH	-Br	-H	-OH	-H	

-OH	-H	-OH	-H	-OH	-Br	-H	-OH	-H	
-OCH$_3$	-OMe	-OCH$_3$	-H	-Cl	-OH	-H	-OH	-H	
-OH	-OH	-OH	-H	-Cl	-OH	-H	-OH	-H	
-OH	-Cl	-OH	-H	-OH	-OH	-H	-OH	-H	
-OH	-Br	-OH	-H	-OH	-OH	-H	-OH	-H	
-OH	-H	-OH	-Br	-OH	-OH	-H	-OH	-H	8BrQ
-OH	-Br	-OH	-Br	-OH	-OH	-H	-OH	-H	DBrQ
-OH	-I	-OH	-I	-OH	-OH	-H	-OH	-H	DIQ
-OH	-Cl	-OH	-Cl	-OH	-OH	-H	-OH	-H	DClQ
-OH	-I	-OH	-H	-OH	-OH	-H	-OH	-H	6IQ
-OH	-H	-OH	-I	-OH	-OH	-H	-OH	-H	8IQ
-OH	-Br	-OH	-CH$_2$N(CH$_3$)$_2$	-OH	-OH	-H	-OH	-H	
-OH	-H	-OH	-CH$_2$NH-(*p*-nitrophenyl)	-OH	-OH	-H	-OH	-H	
-OH	-H	-OH	-CH$_2$N=N-(*p*-nitrophenyl)	-OH	-OH	-H	-OH	-H	
-OH	-H	-OH	-CH$_2$N=N-(2,3-diazolyl)	-OH	-OH	-H	-OH	-H	
-OH	-H	-OH	-CH$_2$NH-(*m*-hydroxyphenyl)	-OH	-OH	-H	-OH	-H	
-OH	-H	-OH	-CH$_2$NH-(4-pyridyl)	-OH	-OH	-H	-OH	-H	
-OH	-H	-OH	-CH$_2$NH(*N*-morpholinyl)	-OH	-OH	-H	-OH	-H	
-OH	-H	-OH	-CH$_2$NH(*N*-phthalimidyl)	-OH	-OH	-H	-OH	-H	
-OH	-H	-OH	-CH$_2$-(*N*-pyrrolidinyl)	-OH	-OH	-H	-OH	-H	
-OH	-H	-OH	-CH$_2$-NH-(*N*-pyrrolidinyl)	-OH	-OH	-H	-OH	-H	
-OH	-H	-OH	-CH$_2$NHCH$_2$(3,4-dioxolephenyl)	-OH	-OH	-H	-OH	-H	
-OH	-H	-OH	-CH$_2$NH-(2,3-diazolyl)	-OH	-OH	-H	-OH	-H	
-OH	-H	-OH	-CH$_2$NHCH$_2$(cyclopropyl)	-OH	-OH	-H	-OH	-H	
-OH	-H	-OH	-CH$_2$NH-(cyclopropyl)	-OH	-OH	-H	-OH	-H	
-OH	-H	-OH	-CH$_2$NH-(cyclobutyl)	-OH	-OH	-H	-OH	-H	
-OH	-H	-OH	-CH$_2$NHCH$_2$(cyclopentyl)	-OH	-OH	-H	-OH	-H	
-OH	-H	-OH	-CH$_2$NH-(2-furanyl)	-OH	-OH	-H	-OH	-H	
-OH	-H	-OH	-CH$_2$NHCH$_2$(cyclohexyl)	-OH	-OH	-H	-OH	-H	
-OH	-H	-OH	-CH$_2$NH-benzyl	-OH	-OH	-H	-OH	-H	
-OH	-H	-OH	-CH$_2$NHCH(COOH)CH$_2$CH$_2$CONH$_2$	-OH	-OH	-H	-OH	-H	
-OH	-H	-OH	- CH$_2$NHCH(COOH)CH$_2$OH	-OH	-OH	-H	-OH	-H	
-OH	-H	-OH	-CH$_2$NHCH(COOH)CH$_2$C(CH$_3$)$_2$	-OH	-OH	-H	-OH	-H	
-OH	-H		-CH$_2$N-CH$_2$-(phenyl)<br>-O-CH$_2$/	-OH	-OH	-H	-OH	-H	
-OH	-H		-CH$_2$N-CH$_2$CH$_3$<br>-O-CH$_2$/	-OH	-OH	-H	-OH	-H	
-OH	-H	/CH$_2$CH$_2$CH(CH$_3$)$_2$<br>R3: -O-CO-CH- NH<br>R4 : -CH$_2$/		-OH	-OH	-H	-OH	-H	
-OH	-H	-OH	-OH	-OH	-OH	-H	-OH	-H	
-OH	-H	-OH	-H	-OH	-OH	-H	-OH	-H	Quercetin
-OH	-OH	-OH	-H	-OH	-OH	-H	-OH	-H	Quercetigetin
-OH	-H	-OH	-H	-OH	-OH	-OH	-OH	-H	Myricetin
-H	-H	-OH	-H	-OH	-OH	-H	-OH	-H	Fisetin
-OH	-H	-OH	-H	-H	-H	-H	-OH	-H	Galangin

-OH	-H	-OH	-H	-OH	-H	-H	-OH	-H	Kampferol
-OAc	-H	-OAc	-H	-OAc	-OAc	-H	-OAc	-H	Pentaacetyl Qurcetin
-OH	-H	-OH	-H	-OH	-H	-H	-OH	-OH	Morin
-OH	-H	-OH	-H	-OH	-OH	-H	-H	-H	Luteolin
-OH	-H	-OH	-H	-OH	-H	-H	-H	-H	Apigenin
-OH	-OH	-OH	-H	-H	-H	-H	-H	-H	Baicalien
-OH	-H	-OH	-H	-H	-H	-H	-H	-H	Chrysin
-OH	-OH	-OH	-H	-OH	-H	-H	-H	-H	Scutellarin
-OH	-H	-OH	-H	-OMe	-OH	-H	-OH	-H	4’-O-methylquercetin
-OMe	-H	-OH	-H	-OH	-OH	-H	-OH	-H	5-O-methylquercetin
-OH	-H	-OH	-H	-OH	-OH	-H	-OMe	-H	3-O-methylquercetin
-OH	-OMe	-OMe	-H	-OMe	-OH	-H	-OMe	-H	Casticin
-OH	-H	-OH	-H	-OH	-OMe	-H	-OH	-H	Isorhamnetin
-OH	-H	-OMe	-H	-OH	-OMe	-H	-OH	-H	Rhamnazin
-OH	-H	-OMe	-H	-OH	-OH	-H	-OH	-H	Rhamnetin
-H	-H	-O-CH ‖ -CH		-H	-H	-H	-OMe	-H	Karajin
-OH	-H	-OH	-OMe	-OH	-OMe	-H	-OH	-H	Limocitrin
-OH	-H	-OH	-OH	-OH	-OH	-H	-OH	-H	Gossypetin
-OH	-Me	-OH	-H	-OH	-OH	-H	-OH	-H	6-methylquercetin
-OH	-H	-OH	-H	-OH	-OH	-H	-O-Glucuronic Acid	-H	Miquelianin
-OH	-H	-OH	-H	-OH	-O-Glucose	-H	-OH	-H	Quercetin-4'-o-glucoside
-OH	-H	-OH	-O-beta-D-glucopyranoide	-OH	-OH	-H	-OH	-H	Gossypin

Table S2 Anthocyanins studied for possible PTPRD PAM activities. Additional non-anthocyanin structures tested but not shown: eriodictyol, hesperetin, (+)-catechin, (-)-catechin, (-)-epicatechin, gallocatechin gallate and epigallocatechin gallate.

**R1**	**R2**	**R3**	**3’**	**4’**	**5’**	**common name**
-H	-H	-H	-H	-OH	-OH	Cyanidin
-H	-H	-H	-OH	-OH	-OH	Delphinidin
-H	-H	-H	-OMe	-OH	-OH	Petunidin
-H	-H	-H	-H	-OH	-OMe	Peonidin
-H	-H	-H	-OMe	-OH	-OMe	Malvidin
-H	-H	-rutinose	-H	-OH	-OH	Keracyanin
-H	-H	-glucose	-OMe	-OH	-OMe	Oenin
-H	-H	-rabinose	-H	-OH	-OH	Cyanadin-3-O-arabinoside
-H	-H	-glucose	-H	-OH	-OH	Cyanadin-3-O-glucoide
